# Early cellular mechanisms of type I interferon-driven susceptibility to tuberculosis

**DOI:** 10.1016/j.cell.2023.11.002

**Published:** 2023-11-28

**Authors:** Dmitri I. Kotov, Ophelia V. Lee, Stefan A. Fattinger, Charlotte A. Langner, Jaresley V. Guillen, Joshua M. Peters, Andres Moon, Eileen M. Burd, Kristen C. Witt, Daniel B. Stetson, David L. Jaye, Bryan D. Bryson, Russell E. Vance

**Affiliations:** 1 Divison of Immunology and Molecular Medicine; 2 Howard Hughes Medical Institute, University of California, Berkeley, Berkeley, CA, 94720, USA; 3 Department of Biological Engineering, Massachusetts Institute of Technology, Cambridge, MA, 02139, USA; 4 Ragon Institute of Mass General, MIT, and Harvard, Cambridge, MA, 02139, USA; 5 Department of Pathology and Laboratory Medicine, Emory University, Atlanta, GA, 30322, USA; 6 Department of Immunology, University of Washington, Seattle, WA, 98195, USA; 7 Lead contact: Russell E. Vance

## Abstract

*Mycobacterium tuberculosis* (*Mtb*) causes 1.6 million deaths annually. Active tuberculosis correlates with a neutrophil-driven type I interferon (IFN) signature, but the cellular mechanisms underlying tuberculosis pathogenesis remain poorly understood. We found interstitial macrophages (IMs) and plasmacytoid dendritic cells (pDCs) are dominant producers of type I IFN during *Mtb* infection in mice and non-human primates, and pDCs localize near human *Mtb* granulomas. Depletion of pDCs reduces *Mtb* burdens, implicating pDCs in tuberculosis pathogenesis. During IFN-driven disease, we observe abundant DNA-containing neutrophil extracellular traps (NETs) described to activate pDCs. Cell type-specific disruption of the type I IFN receptor suggests IFNs act on IMs to inhibit *Mtb* control. Single cell RNA-seq indicates type I IFN-responsive cells are defective in their response to IFNγ, a cytokine critical for *Mtb* control. We propose pDC-derived type I IFNs act on IMs to permit bacterial replication, driving further neutrophil recruitment, and active tuberculosis disease.

## In Brief

Induction of an anti-viral type I interferon response strongly correlates with progression to active tuberculosis disease in humans. Using a tuberculosis-susceptible mouse model, two cell types were identified as the major producers of type I interferon and one as a critical sensor, thereby revealing mechanisms underlying type I interferon-driven susceptibility to tuberculosis.

## Graphical Abstract

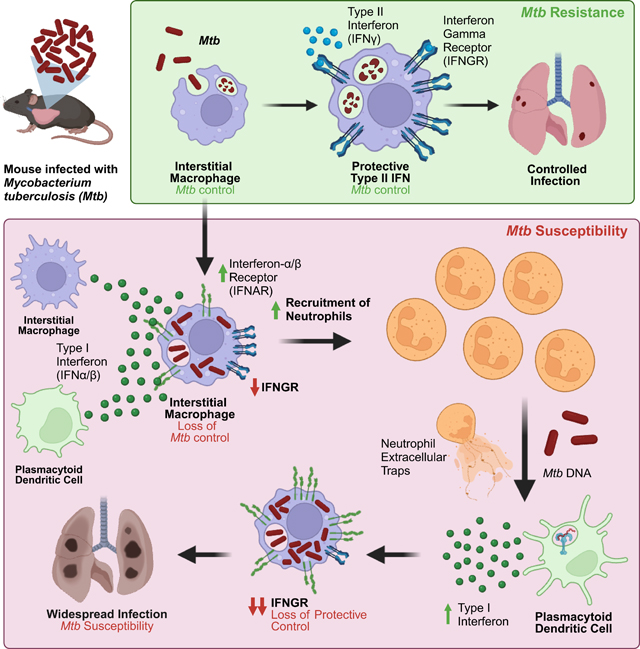

## Introduction

*Mycobacterium tuberculosis* (*Mtb*), the causative agent of tuberculosis disease, caused 1.6 million deaths in 2021^1^. Treatment requires a 4–6 month course of antibiotics, or up to 2 years for increasingly prevalent multi-drug resistant strains. Moreover, the only approved vaccine for *Mtb* has variable or no efficacy in adults^2^. The pathophysiology of tuberculosis remains poorly understood. The mouse model has been used to discover most of the host factors known to control tuberculosis in humans, including tumor necrosis (TNF) factor and interferon-γ^3–5^. Nevertheless, mouse models have been criticized for poorly recapitulating key aspects of human disease^6^.

In humans, active tuberculosis disease is reproducibly associated with the induction of type I interferons (IFNs)^7–9^, a family of cytokines that includes IFNβ and multiple IFNα isoforms. Type I IFNs signal via the type I IFN receptor (IFNAR) to elicit an anti-viral response that overlaps but is insufficient to recapitulate the protective anti-*Mtb* response elicited by IFNγ. Evidence that type I IFNs exacerbate tuberculosis in humans comes from observations that viral infections are associated with worse *Mtb* infection outcomes. For example, influenza infection correlates with an increased risk of death among pulmonary tuberculosis patients, and infants with cytomegalovirus have an increased risk of tuberculosis disease^10–12^. A causal role for type I IFNs in driving human tuberculosis is also supported by the finding that a partial loss-of-function mutation in *IFNAR* is associated with *Mtb* resistance in humans^13^. How type I IFNs drive *Mtb* progression in humans is poorly understood, but one mechanism may involve antagonism of the critical, protective IFNγ response during infection^14,15^.

Mice are able to model the virus-induced susceptibility to *Mtb* seen in humans and have established causality between type I IFN and loss of *Mtb* control. Chronic lymphocytic choriomeningitis virus (LCMV), acute LCMV, pneumonia virus of mice, and influenza A virus all exacerbate tuberculosis infection^16–20^. However, co-infection studies make it challenging to determine the cellular mechanism behind *Mtb* susceptibility, as perturbations such as type I IFN receptor blockade simultaneously impact viral and bacterial control. Therefore, an ideal platform to study type I IFN-driven *Mtb* susceptibility would be a mouse model where *Mtb* infection is itself sufficient to elicit the exacerbated type I IFN response observed in humans with active TB. However, C57BL/6 (B6) mice, the most used model for *Mtb* infection, generate a weak type I IFN response in response to *Mtb* infection; indeed, *Ifnar1* deletion from unmanipulated B6 mice does not consistently impact *Mtb* lung burden^21–24^. Investigators have circumvented this issue by intranasal injection of B6 mice with polyI:C, an IFN-inducing viral mimic^8,25,26^. Such studies demonstrate a convincing causal link between type I IFNs and *Mtb* susceptibility in mice, and have shown that a major detrimental effect of type I IFNs is to impair interleukin-1-dependent control of *Mtb*^26,27^. Despite these advances, it has been challenging to decipher the cellular mechanisms underlying the detrimental effects of type I IFN on bacterial control, and in particular, the cells required to produce or respond to type I IFNs to mediate *Mtb*-susceptibility remain unknown.

Unlike B6 mice, C3H and 129 mice exhibit a type I interferon-driven susceptibility to *Mtb*^28–30^, but there are limited genetic tools in these mouse strains, making mechanistic studies difficult. We recently discovered that congenic B6 mice with the ‘super susceptibility to tuberculosis 1’ region from C3H mice (i.e., B6.*Sst1^S^* mice)^29,30^ are more susceptible to *Mtb* infection (compared to isogenic B6 mice) due to their strong type I IFN response. *Ifnar1* deletion fully rescues the enhanced susceptibility of B6.*Sst1^S^* mice at early timepoints and increases survival^27^. We further identified *Sp140* as the gene within the *Sst1* genetic interval that controls *Mtb* susceptibility, and confirmed that the early *Mtb* susceptibility of *Sp140*^−/−^ mice is also rescued by *Ifnar1* deletion^31^. Our *Sp140*^−/−^ mice were generated on a pure C57BL/6J background, enabling the use of existing genetic tools to dissect tuberculosis pathogenesis.

In this study, we leveraged *Sp140*^−/−^ mice to determine the cellular mechanisms of type I IFN-driven *Mtb* susceptibility. Single cell RNA-sequencing (scRNA-seq) identified interstitial macrophages (IMs) as a major type I IFN producer. A sensitive genetic reporter of type I IFN production corroborated the scRNA-seq findings, and also revealed that plasmacytoid dendritic cells (pDCs) are an additional source of type I IFN during *Mtb* infection. Type I IFN production by pDCs drives disease since pDC depletion rescued the enhanced susceptibility of *Sp140*^−/−^ mice. Loss of bacterial control in *Sp140*^−/−^ mice leads to neutrophil influx and abundant production of DNA-rich neutrophil extracellular traps (NETs), ligands described to promote type I IFN production by pDCs^32,33^. We developed transcriptional signatures that distinguish the response elicited by type I IFN from that elicited by IFNγ. Application of these signatures to our scRNA-seq data indicated that *Mtb*-infected type I IFN-responsive IMs in the lungs have impaired IFNγ responses^15,34,35^. Cell-type specific deletion of *Ifnar1* validated that type I IFN confers susceptibility by acting on IMs. Our findings suggest a model of tuberculosis pathogenesis in which type I IFNs drive an initial loss of bacterial control, possibly by impairing IFNγ responses, that in turn initiates a positive feedback loop of NET production and type I IFN expression by pDCs, leading to uncontrolled bacterial replication and active tuberculosis disease.

## Results

### Myeloid cells harbor Mtb in Sp140^−/−^ mice

**Figure 1.**
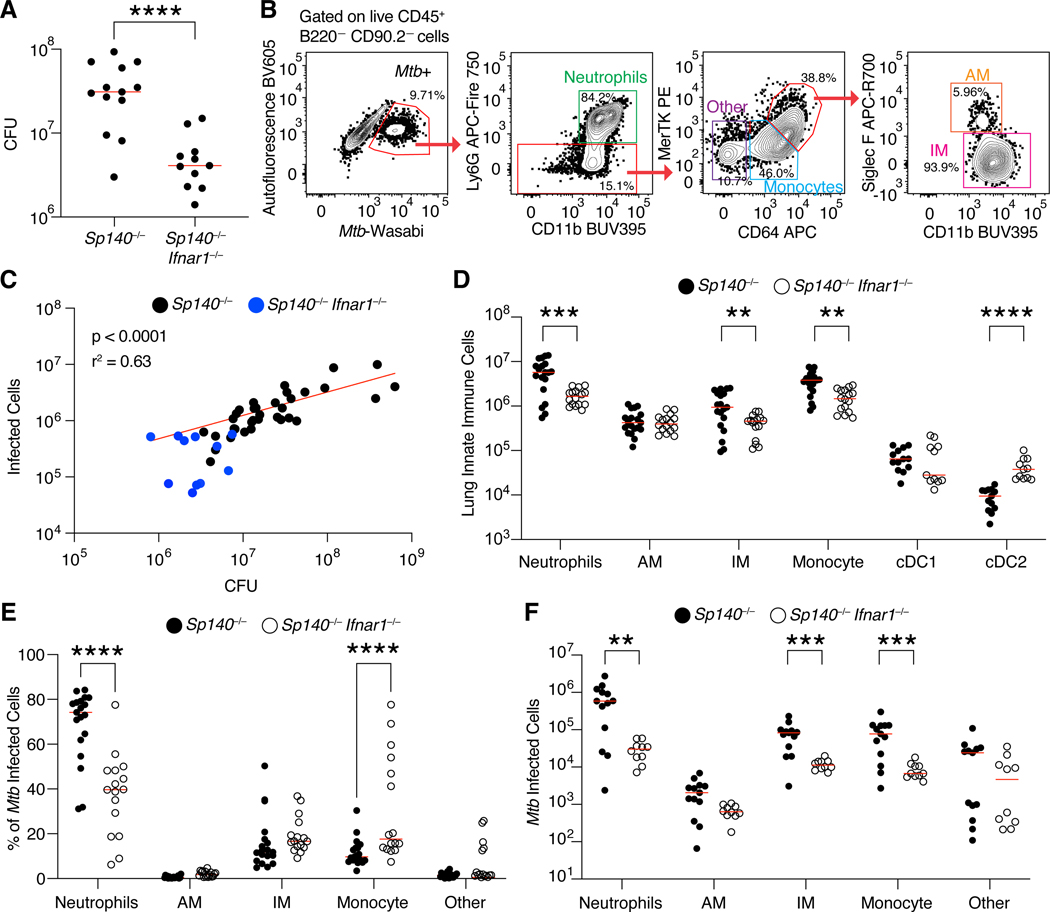
Myeloid cells are the dominant *Mtb* harboring cells in *Sp140*^−/−^ and *Sp140*^−/−^ *Ifnar1*^−/−^ mice. (**A**) Colony forming units (CFU) of *Mtb* in the lungs of *Sp140*^−/−^ (n = 13) and *Sp140*^−/−^ *Ifnar1*^−/−^ (n = 11) mice. (**B**) Representative flow cytometry plots of an *Mtb*-infected *Sp140*^−/−^ mouse lung gated on live CD45^+^B220^−^CD90.2^−^ cells to identify *Mtb*-infected cells, subset into neutrophils (green; Ly6G^+^ CD11b^+^), other cells (purple; Ly6G−CD64−MerTK−), monocytes (blue; Ly6G−CD64^+^MerTK^low^), alveolar macrophages (AMs; orange; Ly6G− CD64^+^MerTK^high^Siglec F^+^), and interstitial macrophages (IMs; pink; Ly6G−CD64^+^MerTK^high^Siglec F−). (**C**) Correlation between infected cell numbers identified by flow cytometry of total lung digests to CFU from the same infected lung (black; *Sp140*^−/−^ and blue; *Sp140*^−/−^*Ifnar1*^−/−^ combined; n = 45). Red line indicates a nonlinear regression. (**D**) Number of innate immune cells by cell type in *Mtb*-infected *Sp140*^−/−^ (n = 19; closed circles) and *Sp140*^−/−^*Ifnar1*^−/−^ lungs (n = 16; open circles). (**E**) Frequency and (**F**) number of immune cell populations of *Mtb*-infected cells in *Sp140*^−/−^ (n = 12–19; closed circles) and *Sp140*^−/−^ *Ifnar1*^−/−^ mice (n = 10–15; open circles). Lungs were analyzed 24–26 days after *Mtb* infection. The bars in (A), (D), (E), and (F) represent the median. Pooled data from two or three independent experiments are shown. A linear regression performed on log transformed data was used to calculate significance and R^2^ for (C). An unpaired t test was used to determine significance for (A), a two-way ANOVA with Sidak’s multiple comparisons test was used to calculate significance for (D), (E), and (F). **p < 0.01, ***p < 0.001, ****p < 0.0001.

As we previously demonstrated, the susceptibility of *Sp140*^−/−^ mice to *Mtb* is driven by type I IFN, and mirrors the correlation between type I IFN and active tuberculosis disease seen in humans. Genetic or antibody-mediated depletion of IFNAR fully rescues the enhanced susceptibility of *Sp140*^−/−^ animals (Fig. 1A)^31^. To better characterize the immune response of *Sp140*^−/−^ mice to *Mtb*, we infected mice with *Mtb* expressing the fluorescent protein Wasabi (*Mtb*-Wasabi)^36^. With this approach, we identified *Mtb*-harboring cells in mouse lungs 25 days post-infection (Fig. 1B). Flow cytometry reliably reported overall lung bacterial burdens as the number of *Mtb*-infected cells detected by flow cytometry correlated with lung *Mtb* CFU (R^2^ = 0.63; Fig. 1C). Infected lungs from *Sp140*^−/−^ animals contained significantly more neutrophils, IMs, and monocytes as compared to *Sp140*^−/−^ *Ifnar1*^−/−^ mice, with no difference in the number of AMs and a reduction in the number of cDC2 (Fig. 1D, Supplementary Fig. 1). Over 90% of infected cells were myeloid cells (Fig. 1B, 1E), in line with previous reports^37,38^. Consistent with their higher abundance in infected *Sp140*^−/−^ lungs, neutrophils comprised a considerably larger percentage and absolute number of the *Mtb*-infected cells in *Sp140*^−/−^ mice compared to *Sp140*–/–*Ifnar1*^−/−^ animals (Fig. 1E, 1F). There were also more infected IMs and monocytes in *Sp140*^−/−^ mice compared to *Sp140*^−/−^ *Ifnar1*^−/−^ animals, in line with the overall increase in these immune populations in the lungs of infected *Sp140*^−/−^ mice (Fig. 1D, 1F). Importantly, we did not observe substantial alterations in the immune compartment of uninfected *Sp140*^−/−^ mice, implying grossly normal hematopoietic development in these mice (Supplemental Fig. 2A-C) in contrast to prior suggestions^39–41^. However, the exact mechanisms causing the infection-induced differences in myeloid cells from *Sp140*^−/−^ mice was unclear, and thus required more in-depth profiling of the myeloid compartment.

### Macrophages and neutrophils exhibit a variety of activation states during Mtb infection

**Figure 2.**
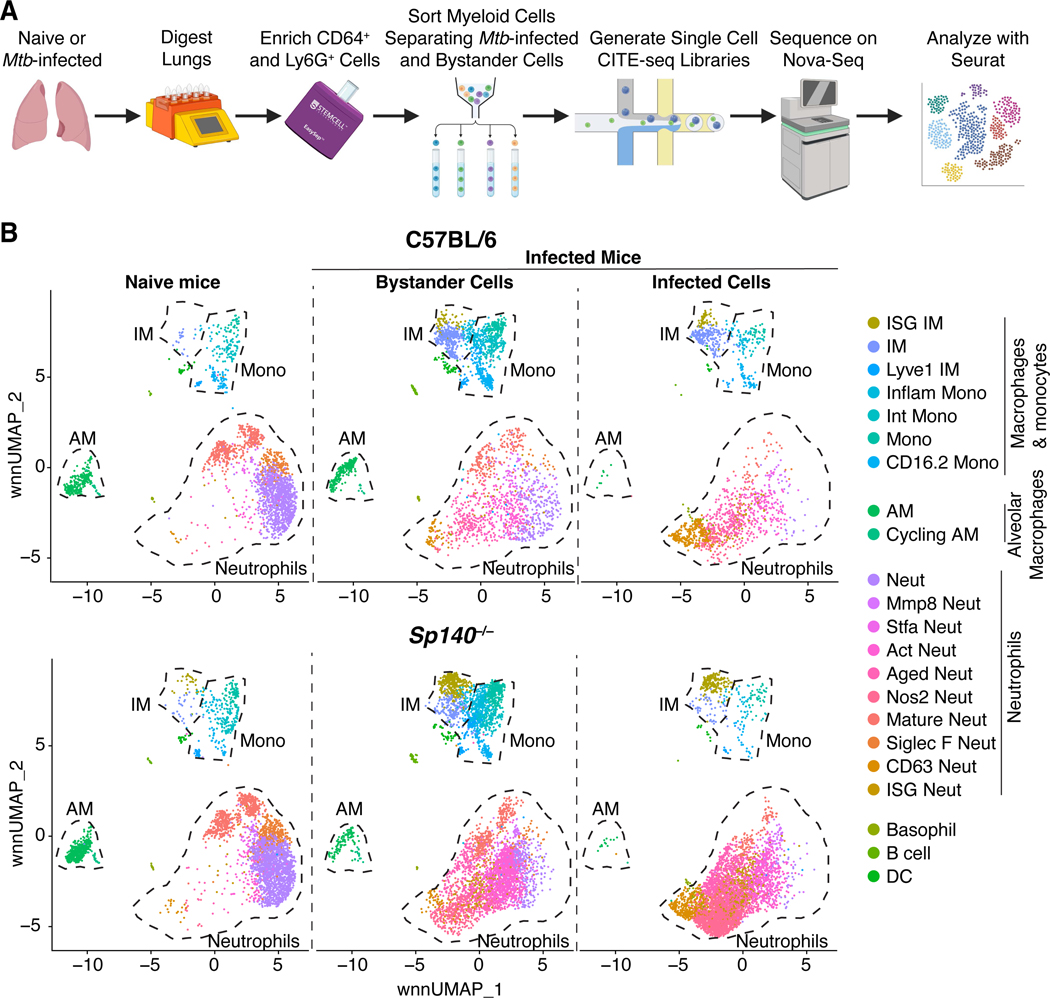
Single cell RNA-sequencing analysis of B6 and *Sp140*^−/−^ myeloid cells from *Mtb*-infected and naïve lungs. CITE-seq was used to integrate transcriptomic and protein expression of single cells, as detailed in Supplementary Fig. 1. (**A**) Model of the processing steps involved in generating the scRNA-seq dataset. (**B**) Unbiased clustering of myeloid cells in B6 and *Sp140*^−/−^ *Mtb*-infected and naïve lungs (n = 10 lungs; n= 20,272 cells) distinguishing cells from naïve mice, the bystander cells from *Mtb*-infected mice, and the *Mtb*-infected cells from *Mtb*-infected mice. Lungs were analyzed 25 days after *Mtb*-Wasabi infection.

To further characterize the *Mtb*-infected myeloid cells in B6 and *Sp140*^−/−^ mice, we performed scRNA-seq on myeloid cells from *Mtb*-infected or uninfected lungs 25 days after infection. For this experiment, CD64^+^ and Ly6G^+^ cells were magnetically enriched, sort purified, and processed for library generation with the 10X Genomics platform (Fig. 2A). Infected and uninfected (bystander) cells from *Mtb*-infected mice were sorted and barcoded separately. mRNA transcripts and protein expression for select lineage markers were simultaneously measured by CITE-seq, allowing for Weighted Nearest Neighbor (WNN) analysis to cluster cells on mRNA and protein expression and WNN uniform manifold approximation and projection (wnnUMAP) reductions for data visualization (Supplementary Fig. 1)^42–46^. The resulting dataset consists of 6,604 B6 and 13,668 *Sp140*^−/−^ cells, almost exclusively consisting of myeloid cells (Fig. 2B). Each cluster is represented in the two datasets, however the proportions of some clusters are altered between genotypes. Most notably, the ratio of IFN stimulated gene (ISG)^+^ IM to ISG– IM was higher in the *Sp140*^−/−^ mice, as expected from the exacerbated type I IFN response in this strain (Fig. 2B). The largest changes in composition were seen when comparing cells from naïve lungs to bystander and *Mtb*-infected cells from *Mtb*-infected lungs (Fig. 2B). For example, AMs are relatively abundant in naïve lungs but are rare among the *Mtb*-infected cells 25 days post-infection, as also seen by flow cytometry (Fig. 1F, 2B). B6 and *Sp140*^−/−^ myeloid cells from uninfected mice were highly transcriptionally similar (Supplemental Fig. 2D-E), confirming that the type I IFN-driven changes in B6 and *Sp140*^−/−^ mice occur after *Mtb* infection.

### Bystander pDCs and IMs are the primary sources of type I IFN during Mtb infection

**Figure 3.**
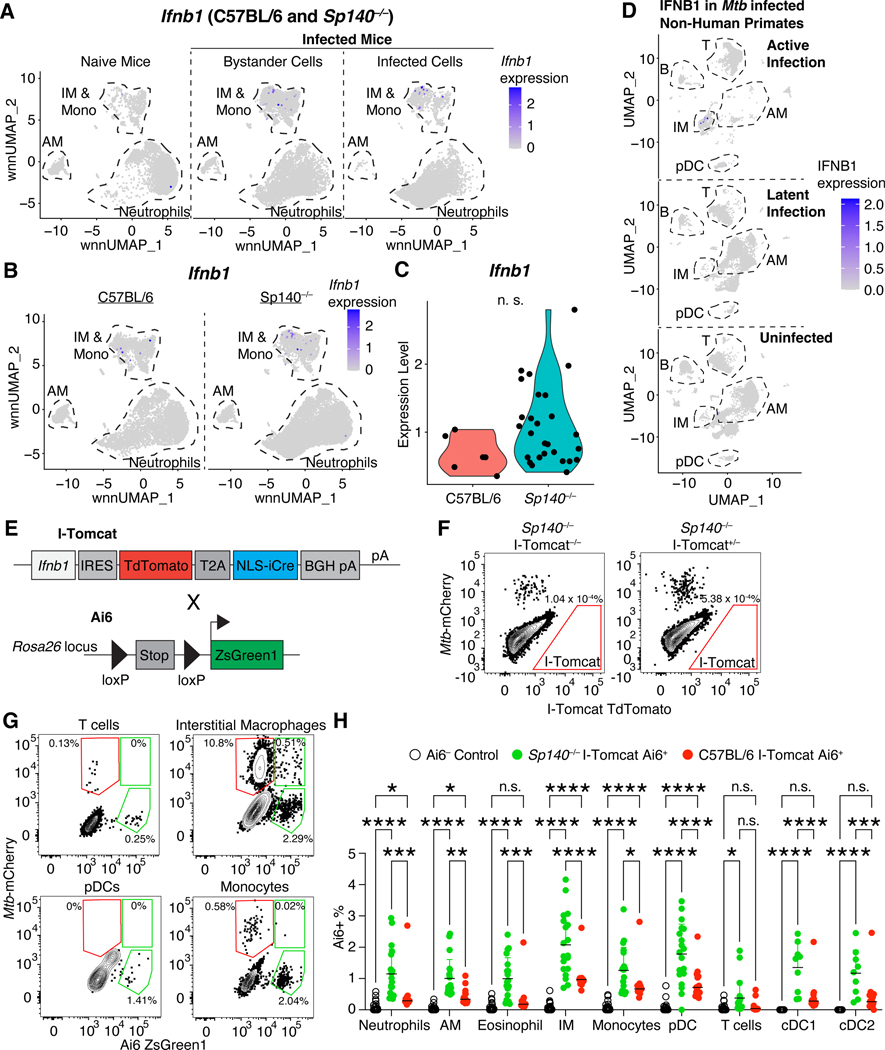
Bystander pDCs, IMs, and monocytes are the primary IFN-β producers in mice and non-human primates. (**A**) *Ifnb1* expression in myeloid cells from naïve mice, bystander myeloid cells from infected mice, and *Mtb*-infected myeloid cells from infected mice (B6 and *Sp140*^−/−^ combined). (**B**) *Ifnb1* expression in myeloid cells (combined infected and bystander) from B6 and *Sp140*^−/−^ mice. (**C**) *Ifnb1* expression in B6 and *Sp140*^−/−^ cells that express *Ifnb1*. (**D**) Analysis of GSE149758 scRNA-seq data from Esaulova E., *et al*. 2021 depicting *IFNB1* expression in cells from non-human primates with active *Mtb* infection, latent *Mtb* infection, or that are uninfected. (**E**) Schematic representation of the genetic structure of I-Tomcat mice and Ai6 mice. (**F**) Representative flow cytometry plot of TdTomato expression in immune cells. (**G**) Representative flow cytometry plots of ZsGreen expression and *Mtb*-mCherry detection in T cells, IMs, pDCs, and monocytes. (**H**) Frequency of Ai6 expressing cells in lung immune cells from Ai6− control (n = 34; open circles), *Sp140*^−/−^ I-Tomcat Ai6 (n = 19; green circles), and I-Tomcat Ai6 (n = 15; red circles) mice. The bars in (H) represent the median. Pooled data from four independent experiments are shown in (H). Lungs were analyzed 25 days after *Mtb* infection. Statistical significance in (C) was calculated by non-parametric Wilcoxon rank sum test with Bonferroni correction and in (H) by one-way ANOVA with Tukey’s multiple comparison test. *p < 0.05, **p < 0.01, ***p < 0.001, ****p < 0.0001.

To determine the cellular mechanism of type I IFN-driven *Mtb* susceptibility, we first sought to identify the type I IFN-producing cells. In general, our scRNA-seq analysis revealed that very few cells were *Ifnb1* positive, which may reflect a lack of sensitivity of scRNA-seq, and/or the transient and stochastic expression pattern of this gene (Fig. 3A)^47–50^. *Mtb* infection resulted in increased expression of *Ifnb1* in infected and bystander mononuclear phagocytes, with a slight bias towards *Ifnb1* production by IMs compared to monocytes, and no production by AMs (Fig. 3A). While there was no major difference in the cell types producing *Ifnb1* between B6 and *Sp140*^−/−^ cells, a greater number and frequency of *Sp140*^−/−^ cells expressed *Ifnb1* (Fig. 3B). Additionally, *Ifnb1*-expressing cells in *Sp140*^−/−^ mice trended towards a higher per cell expression of *Ifnb1* than B6 cells (Fig. 3C). A prior scRNA-seq study of *Mtb*-infected and naïve lungs from non-human primates largely mirrors our findings in mice^51^. Our analyses of these data indicate that IMs were also the dominant *IFNB1*-expressing cells in non-human primates with active tuberculosis, and IMs did not express *IFNB1* in naïve or latently infected lungs (Fig. 3D). These results suggest that mice faithfully recapitulate the *Mtb*-induced type I IFN production seen in non-human primates.

The type I IFN producers identified in the scRNA-seq datasets were validated using an *Ifnb1* genetic reporter called I-Tomcat mice, which express TdTomato and Cre downstream of *Ifnb1* (Fig. 3E, Supplementary Fig. 3). While TdTomato expression was sufficient to identify *Ifnb1* expression by bone marrow-derived macrophages following *in vitro* stimulation with poly I:C (Supplementary Fig. 3), TdTomato^+^ cells were not detected 25 days after *Mtb* infection (Fig. 3F). TdTomato detection was not improved in I-Tomcat homozygous mice, examining an earlier timepoint of 19 days post-infection, or by gating on specific immune populations such as IMs (Supplementary Fig. 3). Even though type I IFN drives *Mtb* susceptibility in *Sp140*^−/−^ mice, it is unclear when type I IFN production occurs (Fig. 1A). Type I IFN production may be an early and/or transient event, which would be missed by analyzing a single timepoint. To address this issue, we crossed I-Tomcat mice with the Ai6 Cre reporter mice (I-Tomcat Ai6) on B6 and *Sp140*^−/−^ backgrounds (Fig. 3E)^52^. In these mice, any cell that has ever expressed *Ifnb1* will constitutively express ZsGreen. *Mtb*-infected I-Tomcat Ai6 mice contained reporter-positive myeloid cells and low background was detected among cell populations that are not expected to express *Ifnb1* (e.g., ~0.1% of T cells were Ai6^+^) (Fig. 3G). Consistent with the scRNA-seq analysis, IMs and monocytes were the primary *Ifnb1*-expressing cells in B6 and *Sp140*^−/−^ mice (Fig. 3H, Supplementary Fig. 3). Interestingly, *Sp140*^−/−^ mice exhibited elevated Ai6^+^ expression frequency in all cell types, suggesting SP140 broadly modulates the sensitivity for inducing *Ifnb1* expression (Fig. 3H). In addition to corroborating the scRNA-seq data, the I-Tomcat mice also identified pDCs as a major type I IFN-producing cell population. Lung pDCs are very rare CD64^–^/Ly6G^–^ cells and were therefore not represented in our scRNA-seq dataset. However, despite their scarcity, pDCs are extremely robust producers of type I IFNs on a per cell basis^53^.

**Figure 4.**
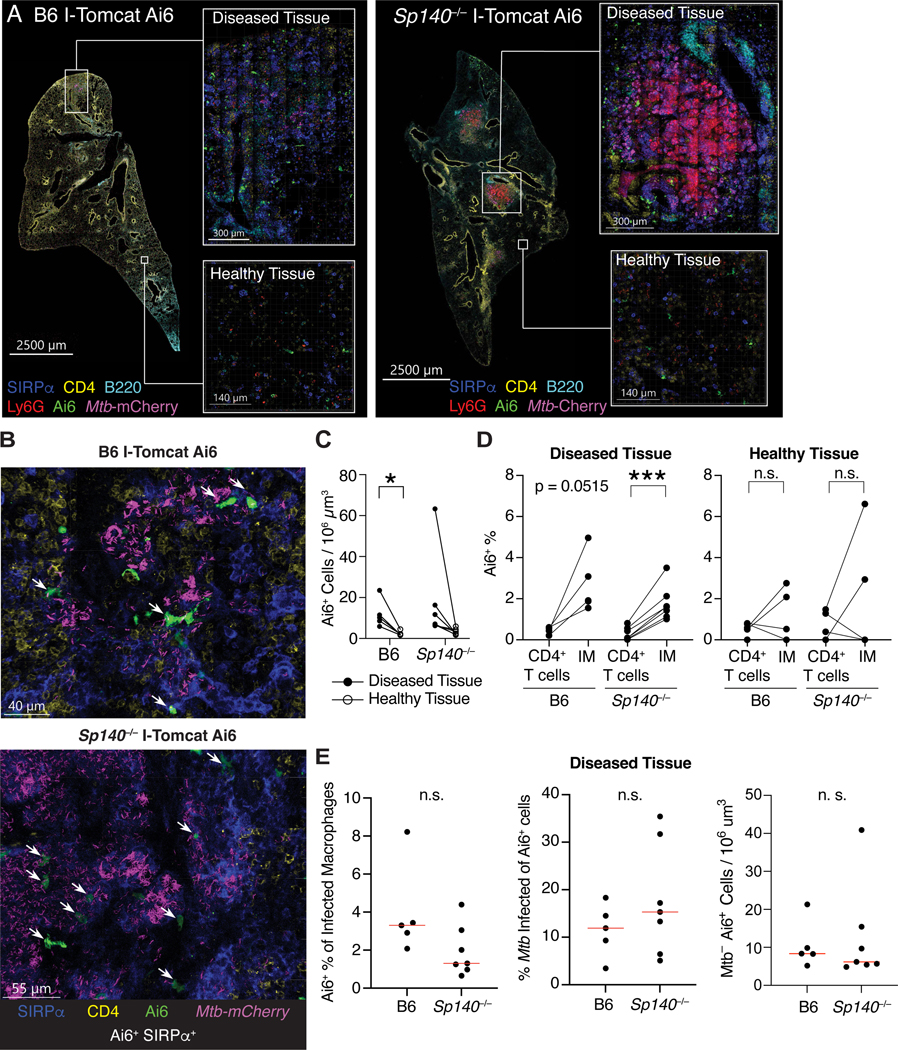
Cells producing IFN-β are enriched in diseased tissue, but only a minority harbor *Mtb*. (**A**) Representative images of *Mtb*-infected I-Tomcat Ai6 and *Sp140*^−/−^ I-Tomcat Ai6 lung sections stained for SIRPɑ (dark blue), CD4 (yellow), B220 (teal), Ly6G (red), Ai6 (green), and *Mtb*-expressed mCherry (magenta). Inset images depict higher magnification of diseased and healthy tissue for both genotypes. (**B**) Representative images of Ai6^+^ cell localization near *Mtb* in the diseased portions of I-Tomcat Ai6 and *Sp140*^−/−^ I-Tomcat Ai6 lungs. Sections were stained with SIRPɑ (dark blue), CD4 (yellow), Ai6 (green), and *Mtb*-expressed mCherry (magenta). White arrows indicate cells co-expressing Ai6 and SIRPɑ. (**C**) Number of Ai6^+^ cells per 10^6^ um^3^ in diseased (closed circle) and healthy tissue (open circle) from B6 (n = 5) and *Sp140*^−/−^ (n = 7) *Mtb*-infected mouse lungs. (**D**) Image quantification of the frequency of Ai6 expression in CD4^+^ T cells and SIRPɑ^+^ IMs in the diseased and healthy tissue of B6 I-Tomcat Ai6 (n = 5) and *Sp140*^−/−^ I-Tomcat Ai6 (n = 7) lungs. (**E**) Image quantification of the frequency of Ai6 expression among *Mtb*-infected macrophages, frequency of *Mtb* infection among Ai6^+^ cells, and number of uninfected Ai6^+^ cells for B6 I-Tomcat Ai6 (n = 5) and *Sp140*^−/−^ I-Tomcat Ai6 (n = 7) lungs. All samples were analyzed 25 days after *Mtb* infection. Pooled data from two independent experiments are shown in (C), (D), and (E). Statistical significance in (C), (D) and (E) was calculated with a paired or unpaired t test. *p < 0.05, ***p < 0.001.

While we expected IMs to be a major type I IFN-producing population given the scRNA-seq results, we were surprised that the majority of the Ai6^+^ IMs were *Mtb*– and most *Mtb*^+^ IMs were Ai6– (Fig. 3G). These results suggest that direct infection of IMs is neither required nor sufficient for IFN-β production. To examine this phenomenon in greater detail, we performed confocal microscopy and histo-cytometry analysis of *Mtb*-infected I-Tomcat Ai6 and *Sp140*^−/−^ I-Tomcat Ai6 lungs^54,55^. While lesions of diseased tissue were clearly identifiable in I-Tomcat Ai6 mice, the size and myeloid cell influx into the diseased tissue were greatly exacerbated in *Sp140*^−/−^ I-Tomcat Ai6 (Fig. 4A). Additionally, Ai6 expressing cells were identifiable throughout the lungs, with an increased propensity to localize in diseased rather than healthy tissue (Fig. 4A–4C). Within diseased tissue, Ai6 expressing cells were primarily located near *Mtb* harboring cells in I-Tomcat Ai6 and *Sp140*^−/−^ I-Tomcat Ai6 lungs (Fig. 4B). Similar to the flow cytometry results, SIRPɑ^+^ cells, which are primarily macrophages in *Mtb*-infected lungs as they are ~100 fold more abundant than SIRPɑ expressing cDC2s, were a major Ai6 expressing cell population (Fig. 1D, 4B, 4D). The SIRPɑ^+^ macrophages expressed Ai6 at a higher frequency than CD4^+^ T cells in the diseased tissue but not healthy tissue (Fig. 3H, 4B, 4D). Direct infection by *Mtb* did not appear to be a major driver of IFN-β expression, as ~2–3% of infected macrophages were Ai6^+^ and ~12–15% of Ai6^+^ cells were *Mtb*-infected, in line with the frequencies seen in IMs by flow cytometry (Fig. 3H, 4E). These results suggest that IM localization to *Mtb* rich regions provides the activating signals required for IFN-β expression, while direct infection of IMs is not required for IFN-β expression.

### pDCs contribute significantly to the susceptibility of Sp140^−/−^ animals to Mtb

**Figure 5.**
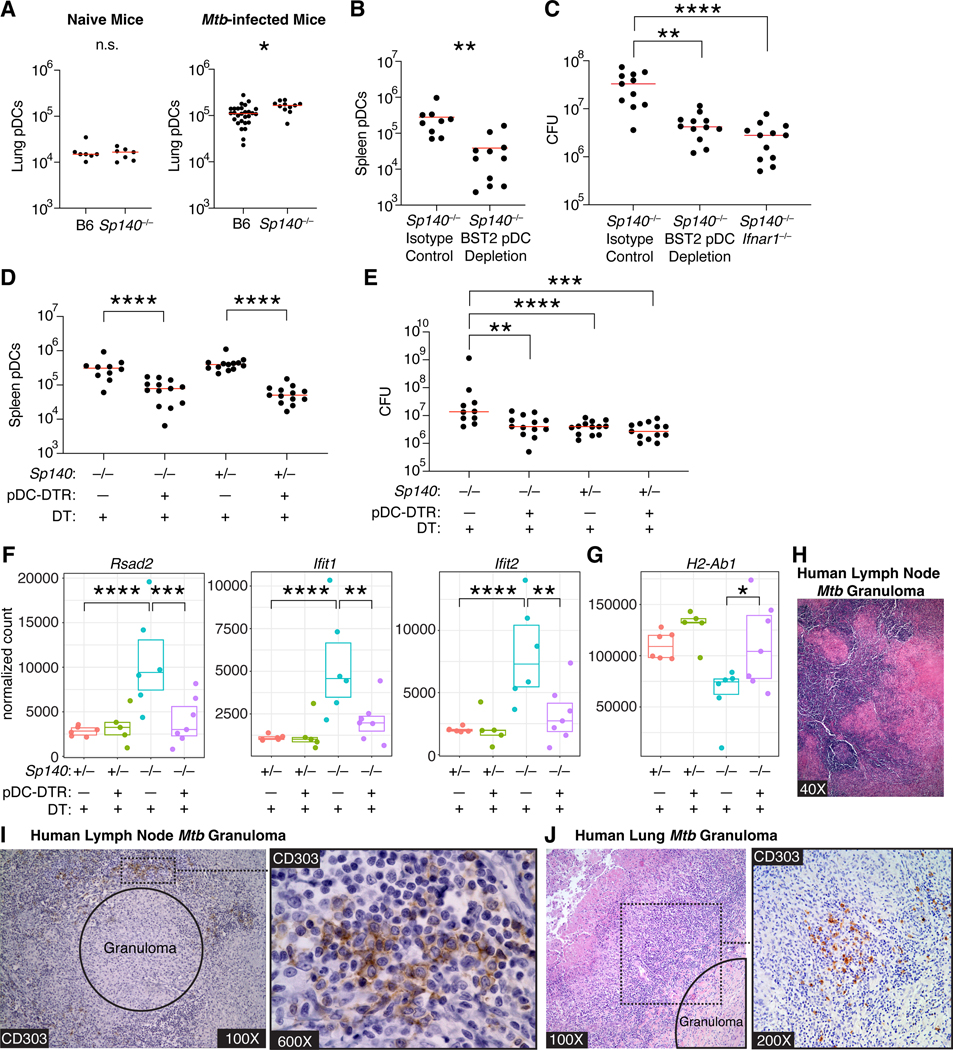
pDC depletion reduces *Mtb* burdens in *Sp140*^−/−^ mice, and pDCs are present in the lymphocytic cuff surrounding granulomas in *Mtb*-infected human lymph nodes and lungs. (**A**) Number of lung pDCs in B6 and *Sp140*^−/−^ mice in naïve (n = 7) and *Mtb*-infected mice (n = 11–28). (**B**) Number of splenic pDCs and (**C**) bacterial burden in *Sp140*^−/−^ mice that received isotype or pDC depleting anti-BST2 antibody from days 12–24 post-infection (n = 9–12). (**D**) Number of splenic pDCs, (**E**) bacterial burden, (**F**) lung expression of *Rsad2*, *Ifit1*, and *Ifit2* as representative type I IFN-stimulated genes, and (**G**) lung expression of *H2-Ab1* as a representative type II IFN-stimulated gene in *Sp140*^−/−^ pDC-DTR mice or *Sp140*^−/−^ mice controls that received DT from days 12 to 24 after infection (n = 10–13 for (D) and (E); n = 5–7 for (F) and (G)). (**H**) Representative hematoxylin and eosin or (**I**) anti-CD303 (brown) and hematoxylin staining on serial sections of *Mtb*-infected human lymph nodes (n = 8). (**J**) Representative hematoxylin and eosin and anti-CD303 (brown) and hematoxylin staining on serial sections of *Mtb*-infected human lung samples (n = 8). Mouse lungs were harvested 25 days post-infection. The bars in (A), (B), (C), (D), and (E) represent the median. Pooled data from two independent experiments are shown in (A), (B), (C), (D), and (E). Statistical significance was calculated by one-way ANOVA with Tukey’s multiple comparison test for (C), (D), and (E) and by an unpaired t test for (A) and (B). *p < 0.05, **p < 0.01, ***p < 0.001, ****p < 0.0001.

While pDCs have a well-established role in anti-viral immunity in the lung, limited work has assessed their contribution during *Mtb* infection^56,57^. pDCs may have been previously overlooked because of their scarcity in the lung. Indeed, we observe only ~20,000 pDCs in the lungs of naïve B6 and *Sp140*^−/−^ mice, but this number increases 10-fold following *Mtb* infection and is modestly but significantly higher in *Mtb*-infected *Sp140*^−/−^ than B6 mice (Fig. 5A). Despite their scarcity, pDCs can have major effects due to the extremely high levels of interferons produced per cell^53^. Consistent with a role for pDCs during *Mtb* infection in mice, Khader and colleagues described the presence of pDCs in lungs of non-human primates with active pulmonary TB^51^. However, the lack of genetic tools in non-human primates precluded functional studies of pDCs during TB. Therefore, we assessed whether pDCs affect *Mtb* control using our experimentally tractable mouse model and an anti-BST2 antibody^58–60^. This strategy efficiently depleted pDCs and resulted in a partial rescue of *Mtb* control in *Sp140*^−/−^ mice (Fig. 5B, 5C). However, BST2 is known to be upregulated by cells other than pDCs in inflammatory environments; thus, antibody depletion could have been protective against *Mtb* by depleting non-pDC cells^59^. We therefore also tested the contribution of pDCs by using a genetic pDC depletion strategy by crossing *Sp140*^−/−^ mice with mice expressing the diphtheria toxin receptor (DTR) downstream of the human BDCA2 promoter (pDC-DTR)^61^. We used *Sp140*^+/−^ pDC-DTR littermates as wild-type controls since a single copy of *Sp140* is sufficient to restore *Mtb* control. DT administration efficiently ablated pDCs in *Sp140*^−/−^ and *Sp140*^+/−^ mice, with the depletion specifically affecting pDCs (Fig. 5D, Supplementary Fig. 4). pDC-DTR depletion of pDCs fully rescued bacterial control in *Sp140*^−/−^ mice, while depletion in *Sp140*-sufficient animals did not affect lung bacterial burden, as expected (Fig. 5E). Additionally, pDC-DTR depletion of pDCs in *Sp140*^−/−^ mice reduced expression of type I IFN stimulated genes to the level of *Sp140*-sufficient animals, while rescuing expression of the type II IFN stimulated gene *H2-Ab1* (Fig. 5F, 5G). These results demonstrate a substantial contribution of pDCs in limiting *Mtb* control in animals with a hyper type I IFN response.

We next sought to understand the role of lung pDCs during human *Mtb* infection by analyzing human lung and lymph node biopsies taken from *Mtb* culture-positive patients for the presence of pDCs near *Mtb* granulomas (Fig. 5H). Based on CD303 and CD123 staining, pDCs localized to the lymphocytic cuff surrounding *Mtb* granulomas in human lungs and lymph nodes (Fig. 5I, 5I, Supplementary Fig. 4). Of the 8 patient samples analyzed, 5 lung samples and 7 lymph node samples had pDCs in the same 400× field as an *Mtb* granuloma (Supplementary Table 1). The majority of the pDCs in the lung samples were distributed as individual cells, while lymph node pDCs were primarily grouped together in clusters of over 20 cells or scattered individually (Supplementary Table 1). These results demonstrate that pDCs, which are overall a rare cell type, cluster near *Mtb*-infected cells in granulomas in human lung and lymph nodes during infection. These results, along with our results in mice and previous studies in non-human primates^51^, implicate pDCs as a plausible source of type I IFN that drives active tuberculosis in humans.

**Figure 6.**
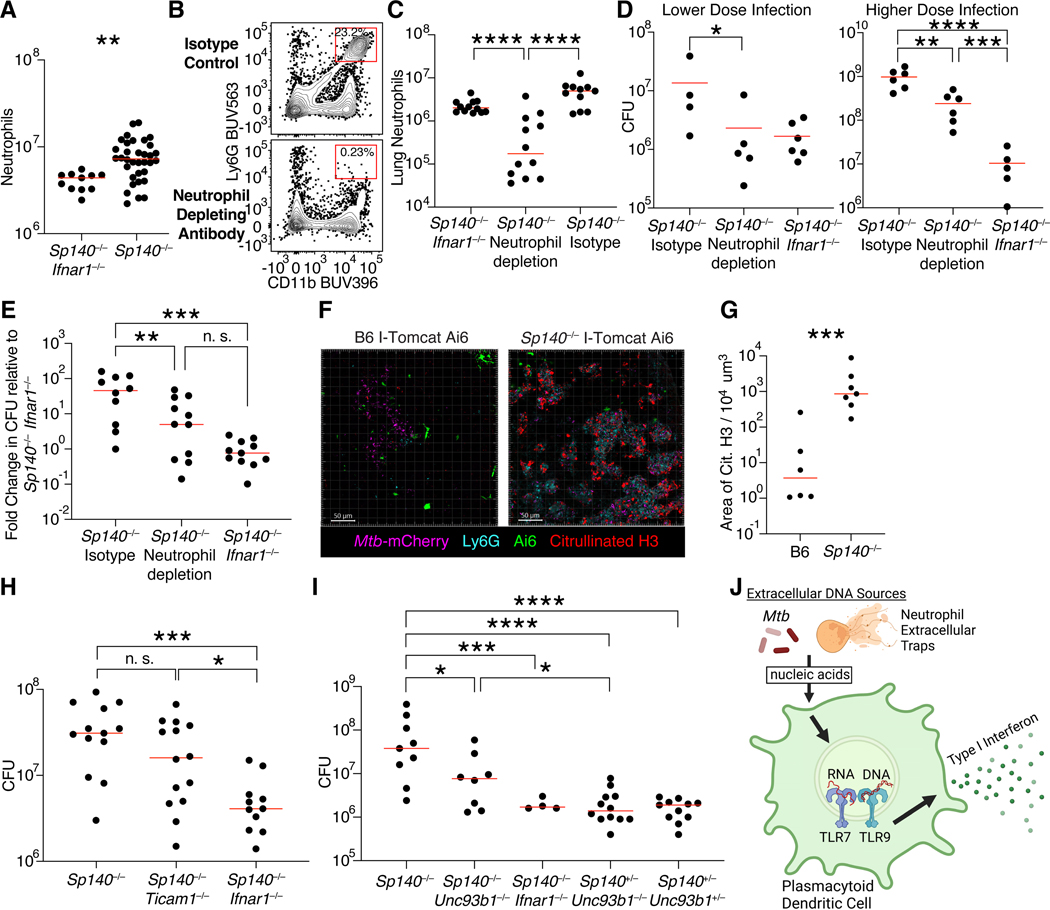
Role of neutrophils, neutrophil extracellular traps (NETs) and endosomal TLRs during type I IFN-driven *Mtb* pathogenesis. (**A**) Quantification of lung neutrophils in *Mtb*-infected *Sp140*^−/−^ (n = 34) and *Sp140*^−/−^ *Ifnar1*^−/−^ (n = 11) mice. (**B**) Representative flow cytometry plot and (**C**) quantification of lung neutrophils (n = 10–11) as well as (**D**) bacterial burden after lower (n = 4–6) or higher dose (n = 5–6) infection and (**E**) combined normalized bacterial burden (n = 10–11) in *Sp140*^−/−^ mice that received isotype or neutrophil depleting anti-Ly6G clone 1A8 antibody from days 12 to 24 post-infection. (**F**) Representative images and (**G**) quantification of NET production based on citrullinated H3 staining in the diseased portions of I-Tomcat Ai6 and *Sp140*^−/−^ I-Tomcat Ai6 lungs. Sections were stained with citrullinated H3 (red), Ai6 (green), Ly6G (teal), and *Mtb*-expressed mCherry (magenta) (n = 6–7). (**H**) Lung bacterial burden in *Mtb*-infected *Sp140*^−/−^ (n = 13), *Sp140*^−/−^ *Ticam1*^−/−^ (*Ticam1* encodes TRIF; n = 14), and *Sp140*^−/−^ *Ifnar1*^−/−^ mice (n = 11). (**I**) Lung bacterial burden in *Mtb*-infected *Sp140*^−/−^ (n = 9), *Sp140*^−/−^ *Unc93b1*^−/−^ (n = 8), *Sp140*^−/−^ *Ifnar1*^−/−^ (n = 4), *Sp140*^+/−^ *Unc93b1*^−/−^ mice (n = 12), and *Sp140*^+/−^ *Unc93b1*^+/−^ mice (n = 11). (**J**) Model of potential extracellular DNA sources stimulating pDC production of type I IFNs through endosomal TLR signaling. The bars in (A), (C), (D), (E), (G), (H), and (I) represent the median. Lungs were analyzed 25 days after *Mtb* infection. Statistical significance was calculated by one-way ANOVA with Tukey’s multiple comparison test for (E), (H), and (I), by one-tailed unpaired t test for (D), and by two-tailed unpaired t test for (A) and (G). *p < 0.05, **p < 0.01, ***p < 0.001, ****p < 0.0001.

We sought to understand why pDCs contribute to the susceptibility of *Sp140*^−/−^ but not *Sp140*-sufficient animals. pDCs are not directly infected and typically produce type I IFNs after recognition of extracellular-derived nucleic acid ligands. Thus, we examined the availability of ligands that might potentially activate pDCs to produce type I IFNs. We focused on DNA-rich neutrophil extracellular traps (NETs) as a potential pDC-activating ligand because extracellular DNA can activate pDCs via TLR9, and NETs have been described as a stimulus for type I interferon production by pDCs in mice and humans in the context of autoimmunity^32,33,62^. Additionally, another *Mtb* susceptible mouse model with a hyper type I interferon response identified the presence of NETs in the lungs of susceptible mice and humans with active *Mtb* disease^63^. We found a 5-fold enrichment in lung neutrophils in *Mtb*-infected *Sp140*^−/−^ relative to *Sp140*^−/−^ *Ifnar1*^−/−^ mice (Fig. 1D, 6A). Additionally, neutrophil depletion partially rescued the susceptibility of *Sp140*^−/−^ mice at both lower and higher bacterial burdens (Fig. 6B-E). Given the importance of neutrophils, we assessed NET production in *Sp140*^−/−^ and B6 mice by staining for citrullinated H3 in the lungs of *Mtb*-infected mice (Fig. 6F). *Sp140*^−/−^ mice had over a 100-fold increase in NET staining as compared to B6 animals, indicating that the lungs of *Sp140*^−/−^ mice harbor substantially more ligand to activate type I interferon production by pDCs as compared to wild-type hosts (Fig. 6G). As NETs are a source of nucleic acids, we hypothesized that pDCs would sense the NETs through endosomal TLRs. In line with this prediction, deletion of *Unc93b1*, which is an essential chaperone required for TLR7 and TLR9 function, partially rescued the *Mtb* susceptibility of *Sp140*^−/−^ mice (Fig. 6H). By contrast, deletion of *Ticam1,* which encodes for TRIF, an adapter molecule critical for type I IFN production downstream of TLR3 and TLR4, had no effect on bacterial control in *Sp140*^−/−^ mice (Fig. 6I). Together, these data suggest a model in which extracellular nucleic acid, potentially from NETs, is sensed by endosomal TLRs triggering pDC production of type I IFNs (Fig. 6J).

### Neutrophils and IMs are the major sensors of type I IFNs during Mtb infection

**Figure 7.**
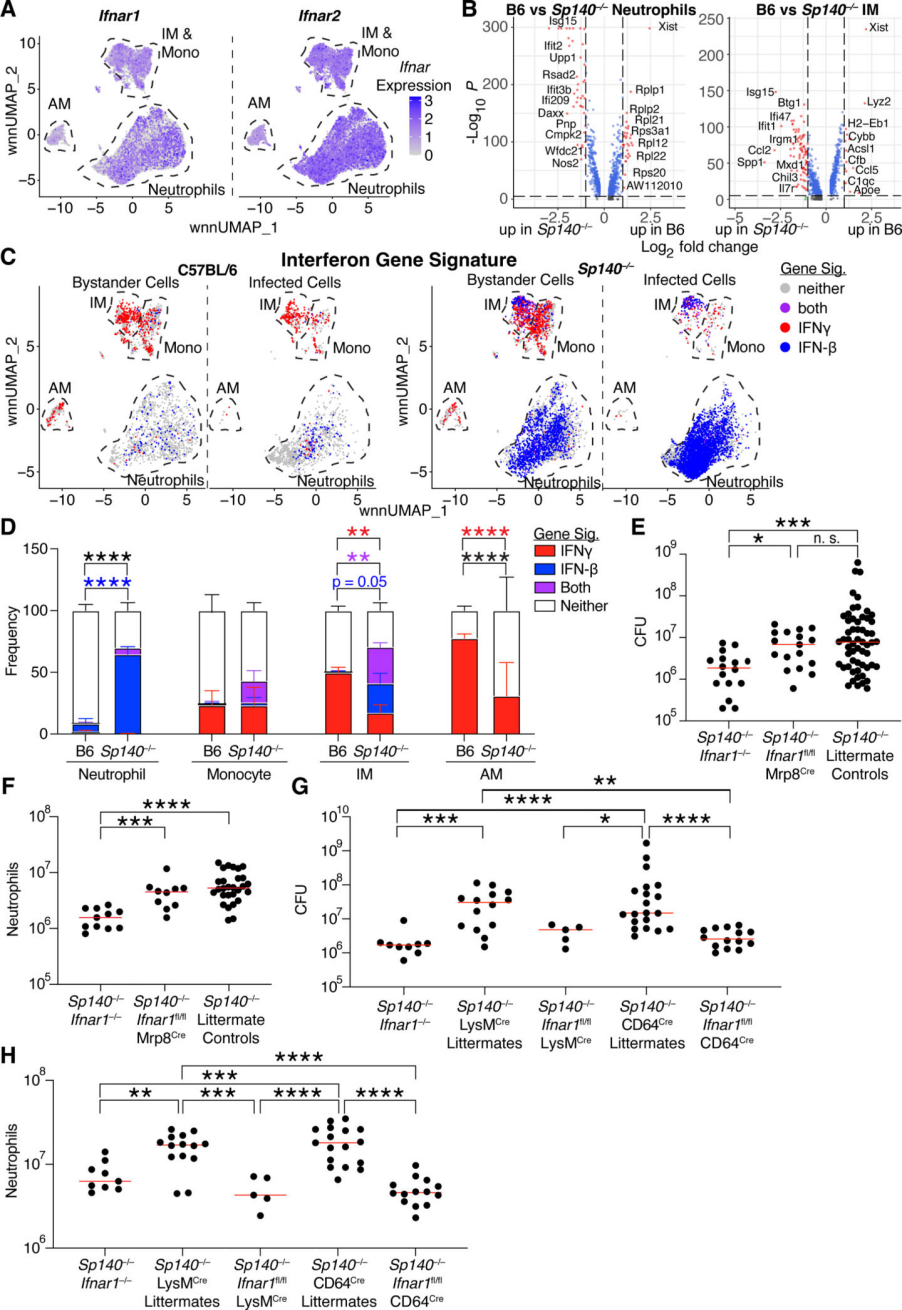
Macrophage recognition of type I IFN drives *Mtb* susceptibility of *Sp140*^−/−^ mice. (**A**) *Ifnar1* and *Ifnar2* mRNA expression in innate immune cells from *Mtb*-infected lungs (B6 and *Sp140*^−/−^ combined). (**B**) Differentially expressed genes comparing B6 and *Sp140*^−/−^ neutrophils and IMs, with higher log fold change indicating greater expression in B6. (**C**) Bystander, and *Mtb*-infected lung myeloid cells from B6 and *Sp140*^−/−^ mice classified by their responsiveness to IFNγ (red), type I IFN (blue), both (purple), or neither (grey). (**D**) Graph of neutrophils, monocytes, IMs, and AMs frequencies from *Mtb*-infected B6 (n = 3) and *Sp140*^−/−^ (n = 3) lungs that are responsive to IFNγ (red), type I IFN (blue), both (purple), or neither (white). (**E**) Lung bacterial burden and (**F**) neutrophils in *Mtb*-infected *Sp140*^−/−^ *Ifnar1*^−/−^ (n = 16), *Sp140*^−/−^ *Ifnar1*^fl/fl^ Mrp8^cre^ (n = 18), and *Sp140*^−/−^ littermate control (n = 57) mice. (**G**) Lung bacterial burden and (**H**) neutrophils in *Mtb*-infected *Sp140*^−/−^ *Ifnar1*^−/−^ (n = 9), *Sp140*^−/−^ LysM^cre^ littermate control (n = 14), *Sp140*^−/−^ *Ifnar1*^fl/fl^ LysM^cre^ (n = 5), *Sp140*^−/−^ CD64^cre^ littermate control (n = 20), and *Sp140*^−/−^ *Ifnar1*^fl/fl^ CD64^cre^ (n = 14) mice. The bars in (E-H) represent the median. Lungs were analyzed 24–26 days after *Mtb* infection. Pooled data from two-three independent experiments are shown. Statistical significance in (B) was calculated by non-parametric Wilcoxon rank sum test with Bonferroni correction, by one-way ANOVA with Tukey’s multiple comparison test for (E-H), and by two-way ANOVA with Tukey’s multiple comparisons test in (D). *p < 0.05, **p < 0.01, ***p < 0.001, ****p < 0.0001.

Having identified pDCs, IMs, and monocytes as the main cells producing type I IFN during *Mtb* infection, we next sought to identify the cells responding to type I IFN. As expected, IFNAR was uniformly expressed by all lung myeloid cells, and was therefore not informative for identifying IFN responsive cells (Fig. 7A)^64^. However, comparing differentially expressed genes in B6 and *Sp140*^−/−^ neutrophils and IMs showed a clear induction of IFN stimulated genes in cells from *Sp140*^−/−^ over B6 animals (Fig. 7B). A major complication is that many genes induced by type I IFN are also induced by type II IFN (IFNγ), and most existing studies do not distinguish the two. Therefore, we sought to develop type I IFN-specific and IFNγ-specific transcriptional signatures. RNA-sequencing analysis of human macrophages and mouse bone marrow-derived macrophages stimulated with IFNγ, IFN-β, TNF, transforming growth factor-β, or nothing were used to define cytokine-induced genes (Supplementary Fig. 5, 6)^65^. Similar gene families were preferentially upregulated by type I or II IFNs in human and mouse macrophages. Genes included in the signatures for type I or II IFN were not strongly induced by a panel of other cytokines (Supplementary Fig. 5). We next applied the mouse gene signatures to our mouse lung myeloid scRNA-seq dataset. The strength of signature expression in naïve mice was used as the threshold for classifying cells as responding to IFNγ or type I IFN (Supplementary Fig. 6). As expected, naïve mice had very few cells responding to either cytokine, while bystander and *Mtb*-infected cells responded strongly to type I and/or II IFNs (Fig. 7C, Supplementary Fig. 6). Interestingly, the type I IFN response was limited to IMs and neutrophils, even though monocytes and AMs were responsive to IFNγ. Potentially, differences in the localization of these cells could explain their differences in cytokine responsiveness. As expected, *Mtb*-infected neutrophils and IMs from *Sp140*^−/−^ mice exhibited a significant increase in type I IFN signaling relative to cells from B6 lungs (Fig. 7D). Consistent with considerable prior work demonstrating that type I IFNs impair responsiveness to IFNγ^15,34,35,66^, the *Sp140*^−/−^ mice harbored a distinct population of infected IMs that exhibited the signature of type I IFN-responsiveness but lacked the signature of IFNγ responsiveness (note the distinct population of blue IMs among the *Mtb*-infected cells in Fig. 7C, 7D). The reduction in IFNγ signaling in *Mtb*-infected IMs in *Sp140*^−/−^ mice correlated with reduced IFNγ receptor 1 expression on these cells in *Sp140*^−/−^ relative to B6 mice (Supplementary Fig. 7). This reduced IFNγ receptor 1 expression also correlated with increased type I IFN stimulated genes and reduced type II IFN stimulated genes (Supplementary Fig. 7). Since IFNγ is critical for *Mtb* control, these results suggest that type I IFN impairs *Mtb* control at least in part by opening a niche of susceptible IMs that fail to respond to IFNγ.

As neutrophils and IMs were the primary sensors of type I IFN, these cell types were tested as potential mediators of type I IFN-driven susceptibility in *Sp140*^−/−^ mice. Neutrophil-specific deletion of *Ifnar1* was insufficient to rescue the *Mtb* susceptibility or the increase in lung neutrophils exhibited by *Sp140*^−/−^ mice (Fig. 7E, 7F). Conversely, deletion of *Ifnar1* expression on myeloid cells with LysM^Cre^ *Ifnar1*^fl/fl^ mice, or specifically in macrophages with CD64^Cre^ *Ifnar1*^fl/fl^ mice, rescued bacterial control and reduced lung neutrophil numbers to the same extent as global *Ifnar1* deletion (Fig. 7G, 7H). Taken together, these results are consistent with a model in which type I IFNs act on IMs to inhibit IFNγ signaling in these cells, thereby reducing their ability to restrict *Mtb* growth.

## Discussion

The dominant gene signature identified in humans with active tuberculosis disease is a type I IFN signature^7,8,67,68^. Type I IFNs are critical for effective anti-viral immunity^67^ but impair *Mtb* control in human and animal models^7,8,26,27,27,28^. For example, viral co-infections or chemical interferon inducers exacerbate *Mtb* disease in mice^16–20^. Interestingly, although type I IFN and IFNγ induce a highly overlapping set of target genes (Supplementary Fig. 5), type I IFNs promote *Mtb* disease while IFNγ potently protects against mycobacterial infections in mice^5^ and humans^8,69–72^. Indeed, type I IFNs can exacerbate bacterial infections in mice and humans by directly antagonizing IFNγ signaling^15,34,35^. The underlying mechanism is poorly understood but may in part be due to downregulation of the IFNγ receptor^34,35^. Type I IFNs can also impair IL-1 signaling, an additional pathway critical for *Mtb* control, through the induction of IL-1 receptor antagonist and eicosanoid imbalance^24,26,27,73^. Thus, the antagonism of protective IFNγ and IL-1 responses by type I IFNs may be a key driver of progression to active tuberculosis. Therefore, we sought a genetically tractable model of *Mtb* infection to establish the cellular mechanisms by which type I IFN drives *Mtb* susceptibility.

The commonly used B6 mouse model does not exhibit a strong type I IFN response after *Mtb* infection^23,27,31^. Consistent with the modest type I IFN response of B6 mice, *Ifnar1* deletion on the B6 background does not reliably impact survival or lung bacterial burden after *Mtb* infection^21–24,27^. Therefore, we sought a different mouse model that recapitulated two key aspects of human disease: the hyper type I IFN response, and the accompanying neutrophilic inflammation^7,74,75^. Previously, we identified B6.*Sst1*S mice as a mouse model that exhibits type I IFN-driven susceptibility to *Mtb* infection^27^. We then demonstrated that the absence of *Sp140* in B6.*Sst1*^S^ mice explains their susceptibility to *Mtb*^31^. SP140 is a member of the Speckled Protein family of epigenetic readers and is widely expressed in leukocytes^76^. *Sp140*-deficient macrophages have been hypothesized to exhibit inherent defects in bacterial control causing increased susceptibility to dextran sulfate sodium-induced colitis^39,40^. However, an inherent defect in bacterial control is not evident during *Mtb* infection, as *Sp140*^−/−^ mice lacking *Ifnar1* restrict *Mtb* as well as B6 animals 25 days after infection^31^, a time point at which macrophages are critical for *Mtb* control^38^. This result suggests that the early susceptibility of *Sp140*^−/−^ mice is due to their strong type I IFN response rather than an inherent defect in bacterial killing by macrophages. In addition to their hyper type I IFN response, *Sp140*^−/−^ mice have more lung neutrophils after *Mtb* infection than *Sp140*^−/−^*Ifnar1*^−/−^ animals (Fig. 1D, 7B, 7D). Therefore, *Sp140*^−/−^ mice recapitulate the fundamental type I IFN and neutrophilic character of human active *Mtb* disease, and can serve as a platform for understanding the cellular mechanism of type I IFN-driven *Mtb* susceptibility.

*Sp140*^−/−^ mice provide an ideal model of the aberrant type I IFN response, as they are on a pure B6 genetic background, and do not require repeated administration of TLR agonists, viral co-infection, or other perturbations of the innate immune system for type I IFN production^16,17,25,26,63^. Other groups have also modeled the type I IFN response by infecting B6 mice with a lineage 2 clinical *Mtb* strain, such as HN878^77,78^. However, *Ifnar1* deletion had no impact on survival or bacterial control at early time points in B6 mice infected with HN878, unlike *Sp140*^−/−^ mice infected with *Mtb* Erdman^31,79,80^. Thus, we believe the *Sp140*^−/−^ mouse model recapitulates the hyper type I IFN response exhibited by humans, and provides a tool to study the mechanistic basis of the aberrant type I IFN response.

We used *Sp140*^−/−^ mice to identify the type I IFN producers and responders that mediate *Mtb* disease. Flow cytometry and imaging of I-Tomcat Ai6 *Ifnb1* reporter mice identified IMs and pDCs as the major IFN-β producers during *Mtb* infection (Figure 3, 4). Imaging provided insight into why these cells expressed type I IFN, as the frequency of type I IFN-expressing macrophages was enriched relative to CD4^+^ T cells in diseased tissue but not in healthy tissue. This result suggests that proximity to *Mtb* dictates access to activating signals required to induce IFN-β expression by macrophages. However, most IFN-β expressing IMs were not infected with *Mtb*, and most infected IMs were not *Ifnb1* or reporter positive, indicating direct infection is insufficient and may not be the main driver of type I IFN expression *in vivo*. *In vitro* studies have shown that bone marrow-derived macrophages infected with *Mtb* induce type I IFN via the cytosolic DNA-sensing cGAS-STING pathway. However, this pathway does not appear to play a major role *in vivo*^27,81–84^. Instead, our results suggest that uninfected bystander cells responding to extracellular ligands may be the primary producers of type I IFN during *Mtb* infection. Consistent with this hypothesis, we found that mice lacking *Unc93b1*, a chaperone required for TLRs that sense extracellular nucleic acids, exhibit enhanced control of *Mtb* (Figure 6I). Of note, pDCs, which we found to be important type I IFN producers during *Mtb* infection, are robust producers of type I IFN after TLR7/9 sensing of exogenous nucleic acids^58,85,86^.

Expression of type I IFNs by pDCs during *Mtb* infection was particularly noteworthy as limited work exists on the effect of pDCs on *Mtb* control. Production of type I IFNs by pDCs is important for control of viral infections^56,57,61^. pDCs also demonstrate a protective function against bacterial infections such as *Citrobacter rodentium*, *Chlamydia pneumoniae*, and *Klebsiella pneumoniae*^87–90^. However, the contribution of pDCs during *Mtb* infection remains unclear. Blood pDC numbers were reduced in *Mtb*-infected humans, but lung pDC numbers or function were not assessed^91,92^. In non-human primates, active pulmonary *Mtb* correlated with pDC influx and IFN-responsive macrophages into the lungs of rhesus macaques^51^. The granulomas of *Mtb*-infected cynomolgus macaques also contained pDCs, but the pDC frequency did not correlate with bacterial burdens in the granulomas^93^. A major issue in the studies using NHPs or humans is the difficulty in depleting or otherwise functionally assessing the role of pDCs. To address this limitation, we generated *Sp140*^−/−^ pDC-DTR mice^59,61^. Using these mice, we found that pDCs contribute significantly to the susceptibility of *Sp140*^−/−^ mice (Figure 5). Additionally, we identified pDCs in the lymphocytic cuff surrounding *Mtb* granulomas in human lungs and lymph nodes. Together, these results suggest that type I IFN produced by pDCs drives tuberculosis disease in mice, and is likely conserved across non-human primates and humans.

While pDC depletion rescued *Sp140*^−/−^ mice, it had no impact on bacterial burden in B6 animals. This result was expected given that very few myeloid cells in B6 mice expressed a type I IFN signature and *Ifnar1* deficiency also has only modest effects in the B6 background^23,27^. As pDCs are present in B6 and *Sp140*^−/−^ mice, we speculated that the difference in pDC type I IFN production in these mouse strains could be due in part to differences in the availability of activating ligands. As seen in another *Mtb*-susceptible mouse model^63^, and in *Mtb*-infected human lungs^63^, *Sp140*^−/−^ mice had a significant enrichment in NET production compared to B6 mice. NETs are DNA-rich products of neutrophils and may act as ligands for TLR9 on the pDCs, as described in mouse and human autoimmunity^32,33,62,94^. In support of this hypothesis, neutrophil depletion partially rescued the susceptibility of *Sp140*^−/−^ mice. Given that NET formation was also identified in *Mtb* granulomas in human lung sections^63^, pDC sensing of NETs may contribute to the type I IFN response detected in humans with active *Mtb* disease.

Having defined the cells producing type I IFNs *in vivo* after *Mtb* infection, we then sought to identify the type I IFN responders. To do this, we first developed transcriptional signatures that distinguish the response to type I IFN from the closely related response to IFNγ. Applying these signatures to our scRNA-seq data, we identified neutrophils and IMs as type I IFN sensors. Both IMs and neutrophils harbor *Mtb*, so the effect of type I IFN could be on either or both cell types. In a GM-CSF blockade model of type I IFN-driven *Mtb* susceptibility, neutrophil-specific deletion of *Ifnar1* rescued bacterial control^63^. By contrast, we were unable to detect any rescue of *Sp140*^−/−^ mice when neutrophils lacked *Ifnar1* (Figure 7E-F). Instead we found that deletion of *Ifnar1* on macrophages rescued *Sp140*^−/−^ mouse bacterial control (Figure 7G-H). GM-CSF is critical for maintaining lung alveolar macrophages and enhances the responsiveness of lung monocytes and macrophages to infections, including *Mtb* infection^95–98^. Therefore, it is possible that impairing lung macrophages by GM-CSF blockade shifted the impact of type I IFN on *Mtb* control from macrophages to neutrophils. Our scRNA-seq dataset only contains myeloid cells and therefore cannot address the contribution of type I IFN signaling in other cell types. However, it is likely that type I IFN largely acts through myeloid cells to drive *Mtb* susceptibility, as macrophage-specific deletion of *Ifnar1* rescued *Mtb* control to a similar extent as global *Ifnar1* deficiency. These results suggest that during a GM-CSF sufficient response, type I IFN signaling in macrophages reduces their ability to restrict *Mtb*. The mechanism by which type I IFNs impair *Mtb* clearance by IMs remains unknown. However, we observed that infected IMs in *Sp140*^−/−^ mice express lower levels of IFNγ receptor and IFNγ target genes, and instead primarily exhibited a type I IFN signature, consistent with the exacerbated type I IFN response in these mice (Supplementary Fig. 7). The transcriptional response of *Sp140*^−/−^ IMs contrasted dramatically with that of infected IMs in B6 mice, which control *Mtb* and which exhibited a uniform signature of responsiveness to IFNγ (Figure 7). Given the essential role of IFNγ in controlling *Mtb* in mice and humans, our results suggest that one detrimental effect of type I IFNs is inhibition of IFNγ signaling in infected macrophages^14,34,35^.

Taken together, our results identify the cell types that produce and respond to type I IFNs during IFN-driven tuberculosis disease. We propose that in addition to its previously described role in inhibition of IL-1 signaling, type I IFNs also impair responsiveness to IFNγ, leading to an initial loss of bacterial control. Bacterial replication then leads to neutrophil influx and NET production within the diseased tissue. DNA-rich NETs may be one source of extracellular-derived ligands sensed by endosomal TLRs in pDCs, though cellular RNA or bacterial DNA may be additional sources. Activation of pDCs results in very robust per-cell production of type I IFNs, which we propose acts in a positive feedback loop to further antagonize IFNγ signaling and reduce the ability of IMs to restrict *Mtb* growth. Given the correlations between our results and findings in rhesus macaques and humans with active *Mtb*, we believe that our proposed mechanism of type I IFN-driven loss of *Mtb* control is conserved across species. These findings open the door for the development of therapies targeting NET production or pDC function as host-directed strategies for treating active *Mtb* infection.

### Limitations of the Study

Although we see a strong correlation between NET formation and type I IFN-driven *Mtb* susceptibility, the current study does not directly test the contribution of NETs in this response. Additionally, the present study provides data suggesting that type I IFN signaling correlates with a loss of type II IFN signaling in IMs during *Mtb* infection, but does not directly examine whether *Mtb*-harboring IMs in *Sp140*^−/−^ mice are unable to control *Mtb* infection because of a lack of response to IFNγ. We also limited our studies to ~25 days post-infection, which is an early time point for *Mtb* infection. It is possible that the cellular sources and targets of type I IFN shift to other cell types at later time points in the infection.

## STAR Methods

### RESOURCE AVAILABILITY

#### Lead contact

Further information and requests for resources and reagents should be directed to and will be fulfilled by the lead contact, Russell Vance (rvance@berkeley.edu).

#### Materials availability

Materials used in this study will be provided upon request and available upon publication.

#### Data and code availability

- Raw and processed bulk RNA- and single cell RNA-sequencing data is deposited in the NCBI Gene Expression Omnibus: GSE216023, GSE232827, GSE232922. This paper also analyzes existing, publicly available data, for which the accession numbers are listed in the key resources table.
- Code for bulk RNA- and scRNA-sequencing analysis is available on Github: https://github.com/dmitrikotov/Sp140-Type-I-Inteferon.
- Any additional information required to reanalyze the data reported in this paper is available from the lead contact upon request.

### EXPERIMENTAL MODEL AND STUDY PARTICIPANT DETAILS

#### Animals

Mice were maintained under specific pathogen-free conditions and housed at 23°C with a 12 hour light-dark cycle in accordance with the regulatory standards of the University of California Berkeley Institutional Animal Care and Use Committee. All mice were sex- and age-matched and were 6–12 weeks old at the start of infections. Male and female mice were used in all experiments. Littermate controls were used when possible, as indicated in the figure legends. B6, B6.129S2-Ifnar1^tm1Agt^/Mmjax (*Ifnar1*^−/−^)^99^, B6.Cg-Gt(ROSA)26Sor^tm6(CAG-ZsGreen1)Hze^/J (Ai6)^52^, B6(Cg)-Ifnar1^tm1.1Ees^/J (*Ifnar1*^fl^)^100^, B6.Cg-Tg(S100A8-cre,-EGFP)1Ilw/J (Mrp8^Cre^)^101^, B6.129P2-Lyz2^tm1(cre)Ifo^/J (LysM^Cre^)^102^, C57BL/6J-*Ticam1^Lps2^*/J (*Ticam1*^−/−^)^103^, and B6N.129S4-*Gt(ROSA)26Sor^tm1(FLP1)Dym^*/J (FLPer)^104^ mice were purchased from Jackson Laboratories. C57BL/6N-*Unc93b1^tm1(KOMP)Vlcg^*/Mmucd (*Unc93b1*^−/−^) mice were obtained from the Mutant Mouse Resource and Research Center (MMRRC) at the University of California, Davis, was donated to the MMRRC by the KOMP repository at University of California, Davis, originated from David Valenzuela of Regeneron Pharmaceuticals^105^, and were provided by Gregory Barton at the University of California, Berkeley. *Ifnb1*-Tomato-Cre-pA Terminator (I-Tomcat) mice were generated by Daniel Stetson at the University of Washington as described below. B6-Fcgr1^tm2Ciphe^ (CD64^Cre^)^106^ mice were generated by Bernard Malissen at Centre d’Immunologie de Marseille-Luminy and provided by Yasmine Belkaid at the National Institutes of Health. B6-Tg(CLEC4C-HBEGF)956Cln/J (pDC-DTR)^61^ mice were provided by Adam Lacy-Hulbert at the Benaroya Research Institute. *Sp140*^−/−^ mice were previously generated in-house^31^. *Sp140*^−/−^ *Ifnar1*^−/−^ mice were generated by crossing *Sp140*^−/−^ mice with *Ifnar1*^−/−^ mice in-house. I-Tomcat Ai6 mice were generated by crossing I-Tomcat mice with Ai6 mice, while *Sp140*^−/−^ I-Tomcat Ai6 mice were the result of crossing I-Tomcat mice with *Sp140*^−/−^ and Ai6 mice in-house. *Sp140*^−/−^ mice were crossed in-house with pDC-DTR mice to generate *Sp140*^−/−^ pDC-DTR mice. *Sp140*^−/−^ *Ifnar1*^fl^ LysM^cre^, *Sp140*^−/−^ *Ifnar1*^fl^ Mrp8^Cre^, and *Sp140*^−/−^ *Ifnar1*^fl^ CD64^Cre^ mice were generated by crossing *Sp140*^−/−^ mice with *Ifnar1*^fl^ and LysM^Cre^ or Mrp8^Cre^ or CD64^Cre^ mice in-house.

#### Bacterial strains

*Mtb* strain Erdman was a gift from Sarah Stanley at the University of California, Berkeley. Frozen aliquoted stocks were produced after passing the strain *in vivo* to ensure virulence. *Mtb* expressing Wasabi (*Mtb*-Wasabi) and *Mtb*-mCherry were generated using *Mtb* that had been passaged 2 or fewer times *in vitro*. For these fluorescent strains, *Mtb* was grown in Middlebrook 7H9 liquid medium supplemented with 10% albumin-dextrose-saline, 0.4% glycerol, and 0.05% Tween-80 for 5 days at 37°C. The cells were pelleted and washed in 10% glycerol to remove salt. The bacteria were then electroporated with 1 μg DNA using a 2 mm electroporation cuvette and the following settings: 2500 volts, 1000 Ohms, 25 μF. The pTEC15 plasmid^36^ (Addgene plasmid # 30174), which expresses Wasabi under the control of the Mycobacterium Strong Promoter, was electroporated into *Mtb* to generate *Mtb*-Wasabi^36^. The pMSP12::mCherry plasmid (a gift from Lalita Ramakrishnan, University of Cambridge; Addgene plasmid # 30167), which expresses mCherry under the control of the Mycobacterium Strong Promoter, was electroporated into *Mtb* to generate *Mtb*-mCherry. Following electroporation, bacteria were grown on 7H11 plates supplemented with 10% oleic acid, albumin, dextrose, and catalase, 0.5% glycerol, and either 200 μg / mL Hygromycin for *Mtb*-Wasabi or 50 μg / mL Kanamycin for *Mtb*-mCherry for 3–4 weeks at 37°C. Individual colonies where then propagated in 10 mL inkwell flask cultures using 7H9 medium supplemented with 10% albumin-dextrose-saline, 0.4% glycerol, 0.05% Tween-80, and either Hygromycin for *Mtb*-Wasabi or Kanamycin for *Mtb*-mCherry for 7 days at 37°C. The inkwell cultures were expanded into a 100 mL culture using the same 7H9 supplemented media with antibiotics and cultured for 4–5 days at 37°C. Once the bacteria were in log phase, the culture was filtered with a 5 μm syringe filter and frozen in 1 mL aliquots in 10% glycerol.

### METHOD DETAILS

#### Generation of the I-TOMCAT IFNβ reporter mice

Targeting of C57BL6/J embryonic stem (ES) cells and generation of chimeric mice was performed by Biocytogen. A construct was targeted immediately downstream of the endogenous *Ifnb* stop codon using Cas9 and a gRNA targeting the genomic site TGCAACCACCACTCATTCTGAGG; the underlined sequence represents the protospacer adjacent motif (PAM). The construct included an encephalomyocarditis virus (EMCV) internal ribosome entry site (IRES), coding sequence for the TdTomato red fluorescent protein, a picornavirus T2A “self-cleaving” peptide, a nuclear localization sequence (NLS)-containing Cre recombinase, and a bovine growth hormone (BGH) polyadenylation (pA) sequence that bypasses the endogenous polyadenylation site in the 3’ untranslated region of the *Ifnb* gene.

After the BGH pA sequence, the construct contained a FRT site-flanked phosphoglycerate kinase (PGK)-neomycin resistance cassette. The insertion cassette was flanked by ~2 kilobase homology arms on either side, and a Diptheria Toxin A (DTA) gene at the 3’ end to select against random insertions. Successful targeting of ES cells was confirmed by PCR. After germline transmission, the knockin mice were confirmed by sequencing and then bred to FLPer mice^104^ to remove the FRT-flanked neo cassette.

#### *Mtb* infections

For infection, a frozen aliquot of *Mtb*-Wasabi or *Mtb*-mCherry was diluted in distilled H_2_O, and 9 mL of diluted culture was loaded into the nebulizer of a inhalation exposure system (Glas-Col, Terre Haute, IN) to deliver ~20–100 bacteria per mouse as determined by measuring CFU in lungs 1 day post-infection.

#### Tissue Processing for CFU and Flow cytometry

Mice were harvested at various days post-infection (as described in figure legends) to measure CFUs by plating and innate immune populations by flow cytometry. All lung lobes were harvested into a gentleMACS C tube (Miltenyi Biotec) containing 3 mL of RPMI media with 70 μg / mL of Liberase TM (Roche) and 30 μg / mL of Dnase I (Roche). Samples were processed into chunks using the lung_01 setting on the gentleMACS (Miltenyi Biotec) and incubated for 30 minutes at 37°C. Tissue was then homogenized into a single cell suspension by running the samples on the lung_02 setting on the gentleMACS. The digestion was quenched by adding 2 mL of PBS with 20% Newborn Calf Serum (Thermo Fisher Scientific) and filtered through 70 μm SmartStrainers (Miltenyi Biotec).

For measuring plasmacytoid dendritic cell numbers, spleens were harvested into a 12 well plate with 1 mL of PBS with 2% Newborn Calf Serum and 0.05% sodium azide in each well. The spleens were sandwiched between 100 uM mesh filters and mashed into a single cell suspension with the back of a syringe plunger. The single cell suspensions were filtered through 70 μm SmartStrainers (Miltenyi Biotec).

#### Measuring Bacterial Burden

To measure CFU, 50 μL was taken from each single cell suspension and then serially diluted in phosphate-buffered saline (PBS) with 0.05% Tween-80. Serial dilutions were plated on 7H11 plates supplemented with 10% oleic acid, albumin, dextrose, and catalase and 0.5% glycerol. Colonies were counted after 3 weeks.

#### Flow Cytometry

For flow cytometry, lung single cell suspensions were pelleted and resuspended in 500 μL of PBS with 2% Newborn Calf Serum and 0.05% Sodium azide and 100–150 μL were stained with antibodies for analysis. Spleen single cell suspensions were pelleted and resuspended in 5 mL of PBS with 2% Newborn Calf Serum and 0.05% Sodium azide, of which 50 μL were stained with antibodies. Single cell suspensions were stained for 45 minutes to an hour at room temperature with the following antibodies: TruStain FcX PLUS (S17011E, BioLegend), BUV496-labeled CD45 (30-F11, BD Biosciences), APC-labeled CD64 (X54–5/7.1, BioLegend), BV480-labeled B220 (RA3–6B2, BD Biosciences), BV480-labeled CD90.2 (53–2.1, BD Biosciences), APC-Fire 750-labeled Ly6G (1A8, BioLegend), BUV395-labeled CD11b (M1/70, BD Biosciences), BUV737-labeled CD11c (HL3, BD Biosciences), APC-R700-labeled Siglec F (E50–2440, BD Biosciences), PE-labeled MerTK (DS5MMER, Thermo Fisher Scientific), Super Bright 645-labeled MHC II (M5/114.15.2, Thermo Fisher Scientific), BV421-labeled PD-L1 (MIH5, BD Biosciences), BV711-labeled Ly6C (HK1.4, BioLegend), PE-labeled IFNAR-1 (MAR1–5A3, BioLegend), PE-Cy7-labeled MerTK (DS5MMER, Thermo Fisher Scientific), APC-eFluor 780-labeled CD11b (M1/70, Thermo Fisher Scientific), BUV395-labeled CCRL2 (BZ2E3, BD Biosciences), BUV563-labeled Ly6G (1A8, BD Biosciences), Percp-Cy5.5-labeled B220 (RA3–6B2, BioLegend), BV421-labeled Siglec H (440c, BD Biosciences), BV480-labeled CD19 (1D3, BD Biosciences), BV605-labeled MHC II (M5/114.15.2, BioLegend), BV785-labeled Ly6C (HK1.4, BioLegend), BV605-labeled CD4 (GK1.5, BioLegend), BUV805-labeled CD8ɑ (53–6.7, BD Biosciences), and PE-Cy7-labeled PDCA-1 (eBio927, Thermo Fisher Scientific). All samples also received fixable viability dye (Ghost Dye Violet 510; Tonbo Biosciences), Super Bright Complete Staining Buffer (Thermo Fisher Scientific), and True-Stain Monocyte Blocker (BioLegend) at the same time as the antibodies. Stained samples were fixed with cytofix/cytoperm (BD biosciences) for 20 minutes at room temperature before samples were removed from the BSL3. For intracellular staining, the fixed samples were stained with the following antibodies: PE-Cy7-labeled CD63 (NVG-2, BioLegend), Percp-eFluor 710-labeled iNOS (CXNFT, Thermo Fisher Scientific), BV785-labeled CD206 (C068C2, BioLegend). Cell numbers were calculated by adding fluorescent AccuCheck Counting Beads (Invitrogen) to each sample. Cells were then analyzed on a Fortessa (BD Biosciences) or an Aurora (Cytek) flow cytometer. Data were analyzed with Flowjo version 10 (BD Biosciences).

#### Immune cell depletion

Injections for cell depletions were started 12 days after *Mtb* infection and continued every other day until the mice were harvested 25 days post-infection. Antibody depletion of pDCs was performed by administering 200 μg of anti-BST2 (927, BioXCell) or rat IgG2b isotype control antibody (LTF-2, BioXCell) in 200 μL PBS via intraperitoneal injection^59^. Genetic depletion involved administering 100 ng diphtheria toxin (Millipore Sigma) in 100 μL PBS via intraperitoneal injection into *Sp140*^−/−^ pDC-DTR mice and littermate controls^61^. Antibody depletion of neutrophils was performed by administering 200 μg of anti-Ly6G (1A8, BioXCell) or rat IgG2a isotype control antibody (2A3, BioXCell) in 200 μL PBS via intraperitoneal injection.

#### Immunostaining human lymph nodes and lungs

Human lung and lymph node samples were acquired from the surgical pathology archives of Emory University Hospital with appropriate institutional approval. 8 lung samples and 8 lymph node samples were analyzed. Each sample had been previously culture verified for *Mycobacterium tuberculosis* infection. The samples were formalin-fixed and paraffin-embedded. Sections were cut and stained with anti-CD123 (6h6, Thermo Fisher Scientific), anti-CD303 (124B3.13, Dendritics), or hematoxylin and eosin^107,108^. Primary antibodies were detected by immunoperoxidase staining with the LSAB^+^ System and a standard DAB reaction following manufacturer’s instructions (DakoCytomation). Sections were counterstained with hematoxlyin prior to mounting and microscopy. pDCs were assessed in multiple 400× fields for each section to calculate the frequency of samples containing pDCs and the clustering of pDCs within each sample, defined as either singe cells, loose clusters of 5–20 cells, or tight clusters of more than 20 cells.

#### Confocal microscopy

Confocal Microscopy was performed using a Zeiss LSM 880 laser scanning confocal microscope (Zeiss) equipped with two photomultiplier detectors, a 34-channel GaASP spectral detector system, and a 2-channel AiryScan detector as well as 405, 458, 488, 514, 561, 594, and 633 lasers. 20 μm paraformaldehyde fixed lung sections from *Mtb*-mCherry infected I-Tomcat Ai6 and *Sp140*^−/−^ I-Tomcat Ai6 mice were stained at 4°C overnight with BV421-labeled SIRP_α_ (P84, BD Biosciences), Pacific Blue–labeled B220 (RA3–6B2, BioLegend), eF506-labeled CD4 (RM4–5, BioLegend), and AF647-labeled Ly6G (1A8, BioLegend). Sections detecting the presence of Neutrophil extracellular traps (NETs) were stained with BV421-labeled Ly6G (1A8, BioLegend) and rabbit polyclonal anti-citrullinated histone-H3 (citrulline R2, R8, R17; Abcam), stained with AF488 donkey anti-rabbit secondary (Poly4064, BioLegend). Stained sections were inspected with a 5× air objective to find representative lesions and distal sites and then imaged using a 63× oil immersion objective lens with a numerical aperture of 1.4. For each infected lung, one *Mtb*-heavy lesion image and one distal site image was taken consisting of 20 μm z-stacks acquired at a 1.5 μm step size. For representative NET images, 4×4 tiled images were captured without a z-stack. Additionally, the Zeiss LSM 880 microscope was used to image single color-stained Ultracomp eBeads Plus (Thermo Fisher Scientific) for generating a compensation matrix.

#### Image processing and histo-cytometry analysis

Image analysis was performed using Chrysalis software^55^. Briefly, a compensation matrix was generated by automatic image-based spectral measurements on single color-stained controls in ImageJ by using Generate Compensation Matrix script. This compensation matrix was used to perform linear unmixing on three-dimensional images with Chrysalis. Chrysalis was also used for further image processing, including rescaling data and generating new channels by performing mathematical operations using existing channels. For histo-cytometry analysis, Imaris 9.9.1 (Bitplane) was used for surface creation to digitally identify cells in images based on protein expression^54^. Statistics for the identified cells were exported from Imaris and then imported into FlowJo version 10 (BD Biosciences) for quantitative image analysis.

#### Bulk RNA-seq sample preparation and analysis

RNA-seq of *Mtb*-infected *Sp140*-sufficient and -deficient mouse lungs genetically depleted of pDCs was performed on 20% of each lung single cell suspension, prepared as described for CFU and flow cytometry analysis. Single cell suspensions were preserved in Trizol LS (Thermo Fisher Scientific) and stored at −80°C. The samples were thawed at room temperature for 5 minutes, then 200 μL of chloroform (Thermo Fisher Scientific) was added per 0.75 mL of Trizol LS to each sample and the samples were removed from the BSL3. Samples were centrifuged in Phasemaker tubes (Thermo Fisher Scientific) to isolate total RNA, which was then purified following the RNeasy Micro (Qiagen) protocol starting at the ethanol addition step. Library preparation, sequencing, and read alignment to the mouse genome was performed by Azenta Life Sciences. Libraries were prepared from total RNA using an Illumina kit for Poly(A) selection. Samples were sequenced on an Illumina HiSeq sequencer with paired 150 bp reads to a depth of 20–30 million reads per sample. Sequence reads were trimmed of adapter sequences and low quality nucleotides with Trimmomatic v.0.36^109^ and then mapped to the Mus musculus GRCm38 reference genome with STAR aligner v.2.5.2b^110^. The raw counts were used as input for DESeq2^111^ analysis of differential gene expression.

RNA-seq of cytokine stimulated bone marrow-derived macrophages was performed by differentiating bone marrow from B6 mice in DMEM supplemented with 10% FBS, PenStrep, Glutamin, HEPES, and M-CSF for 7 days then reseeding the cells in a 6-well plate and incubating for 5 days at 37°C and 5% CO_2_. The macrophages were left untreated or stimulated with 10 ng / mL of IFN-β (BioLegend), IFNγ (Abcam), TNF (Peprotech), or transforming growth factor-β (BioLegend) for 6 hours. Cells were lysed with TRK lysis buffer (Omega Bio-Tek) and 2-mercaptoethanol (Thermo Fisher Scientific) followed by total RNA isolation using the E.Z.N.A Total RNA Kit I (Omega Bio-Tek). The library preparation, sequencing, and read alignment to the mouse genome was performed by Azenta Life Sciences as described for the *Mtb*-infected mouse lung samples. Raw counts were used as input for analysis with DESeq2^111^.

#### Sorting Immune Cells for scRNA-seq analysis

3 B6 and 3 *Sp140*^−/−^ mice were infected with less than 100 CFU of *Mtb*-Wasabi bacteria. Lungs from infected animals as well as 2 naïve control mice per genotype were harvested 25 days post-infection and processed as described for CFU and flow cytometry analysis. Single cell suspensions were resuspended in PBS with 2% Newborn Calf Serum and stained with TruStain FcX PLUS (S17011E, BioLegend), APC-labeled Ly6G (1A8, BioLegend), APC-labeled CD64 (X54–5/7.1, BioLegend), and TotalSeq-A-labeled Ly6G (1A8, BioLegend) anti-mouse antibodies on ice for 30 minutes. Myeloid cells were then magnetically enriched using an EasySep APC Positive Selection Kit II (StemCell Technologies) and MojoSort Magnets (BioLegend). All enriched samples were stained with the following panel of TotalSeq-A-labeled anti-mouse antibodies to detect protein expression in the scRNA-seq dataset: Ly6C (HK1.4, BioLegend), CD44 (IM7, BioLegend), CD169 (3D6.112, BioLegend), CD274 (MIH6, BioLegend), Siglec F (S17007L, BioLegend), CSF1R (AFS98, BioLegend), CD11b (M1/70, BioLegend), CD86 (GL-1, BioLegend), MHC II (M5/114.15.2, BioLegend), and CX3CR1 (SA011F11, BioLegend). Each sample was also stained with a unique anti-mouse TotalSeq-A Hashtag antibody (1–6; BioLegend) to allow up to 6 populations to be multiplexed in a single lane on a 10X Genomics Chromium Next GEM Chip^112^. Enriched cells from infected mice were also stained with PE-labeled B220 (RA3–6B2, Tonbo), PE-labeled CD90.2 (30-H12, Tonbo), and BV785-labeled CD45.2 (104, BioLegend) anti-mouse antibodies. Enriched cells from naïve mice were stained with Pacific Blue-labeled B220 (RA3–6B2, BioLegend), Pacific Blue-labeled CD90.2 (53–2.1, BioLegend), PE-labeled F4/80 (BM8, Thermo Fisher Scientific), and BV785-labeled CD45.2 (104, BioLegend) anti-mouse antibodies. All post-enrichment antibody staining was performed on ice for 45 minutes in the presence of True-Stain Monocyte Blocker (BioLegend). Following staining, cells were resuspended in PBS with 2% Newborn Calf Serum and Sytox Blue Dead Cell Stain (Thermo Fisher Scientific). Cells were sort purified using a 100 μm microfluidic sorting chip in a 4 laser SH-800 cell sorter (Sony) on the purity setting. The isolated populations from infected lungs were *Mtb*-infected cells and bystander myeloid cells. Macrophages and a mixture of neutrophils and monocytes were isolated from the naïve lungs and the macrophages were combined with neutrophil/monocyte mixture at a 1:2 ratio for better macrophage representation in the resulting dataset.

#### Single cell RNA: Library generation and sequencing

The scRNA-sequencing libraries were generated using the v3.1 chemistry Chromium Single Cell 3’ Reagent Kit (10X Genomics) largely following the manufacturer protocol with the following minor modifications. Cells were loaded into 3 different lanes on a Chromium Next GEM Chip. Lane 1 was loaded with *Mtb*-infected cells from all 3 B6 and 3 *Sp140*^−/−^ lungs. Lane 2 was loaded with bystander myeloid cells from the 3 infected B6 lungs as well as the myeloid cell mixture from the 2 naïve B6 lungs. Lane 3 was loaded with bystander myeloid cells from the 3 infected *Sp140*^−/−^ lungs as well as the myeloid cell mixture from the 2 naïve *Sp140*^−/−^ lungs. All 3 lanes of the Chromium Next GEM Chip were super-loaded with 29000 cells with a target of 14800 single cells per lane, as hashtag barcoding allows for a lower effective multiplet rate due to the ability to identify most of the multiplets (https://satijalab.org/costpercell/) ^112^. 0.5 U/μL RNaseOUT Recombinant Ribonuclease Inhibitor (Invitrogen) was added to single cell RT master mix during the loading step and 1 μL of ADT and HTO additive primers (0.2 μM stock) were added during the cDNA amplification, as recommended by the CITE-seq and Cell Hashing Protocol (https://cite-seq.com/protocols/)^46^. Following cDNA, ADT, and HTO purification, samples were decontaminated by 2 rounds of centrifugation through 0.2 μM filter microcentrifuge tubes and then removed from the BSL3. Library preparations were completed outside of the BSL3 following the 10X Genomics protocol for the cDNA and the CITE-seq and Cell Hashing Protocol for the ADT and HTO libraries. Quality control of the libraries was performed with a Fragment Analyzer (Agilent). The mRNA, ADT, and HTO libraries were pooled at the following proportions: 85% mRNA, 9% ADT, and 6% HTO. Libraries were sequenced on a NovaSeq 6000 (Illumina) using two lanes of a S1 flow cell and the following cycles read 1 (28 cycles), i7 index (10 cycles), i5 index (10 cycles), read 2 (90 cycles).

#### ScRNA-seq: data processing

Raw sequencing reads for the mRNA libraries were processed into raw count matrices with CellRanger version 4.0.0 (10X Genomics). The ADT and HTO libraries were processed into raw count matrices with CITE-Seq-Count version 1.4.3 (https://hoohm.github.io/CITE-seq-Count/) ^113^. The raw counts for mRNA, ADT, and HTO were analyzed in R^114^ via the RStudio integrated development environment with Seurat v4.1.1^42^ using default settings for normalizing the data, finding variable features, and scaling the data. HTO demultiplexing was performed with the HTODemux function. Data was filtered to only include single cells with between 200 and 4500 genes and less than 5% mitochondrial reads. The resulting datasets were integrated together using 30 dimensions for the FindIntegrationAnchors function and 30 dimensions for the IntegrateData function. The data was then scaled and analyzed by PCA with 30 principal components followed by UMAP analysis with 30 dimensions. Clustering was performed by using 30 dimensions with the FindNeighbors function and a resolution of 0.8 for the FindClusters function.

To improve resolution for clustering innate immune cells, weighted nearest neighbor analysis was used to combine the protein data (ADTs) and the mRNA data when clustering cells. For this analysis, variable ADT features were identified and then normalized using centered log ratio transformation and a margin of 2. The normalized ADT data was then scaled and analyzed by PCA. The ADT and mRNA data was then combined with the FindMultiModalNeighbors function using 30 dimensions for the mRNA and 10 for the protein. The resulting dataset was analyzed by UMAP and clusters were identified with the FindClusters function using algorithm 3 and a resolution of 1.5^44^. Tidyverse^115^, EnhancedVolcano^116^, and various Seurat functions were used for plotting the scRNA-seq data.

#### Type I IFN and IFNγ Gene Signature Analysis

For generating the type I IFN and IFNγ gene signatures, we utilized a published RNA-seq dataset (GEO: GSE20251) of primary human macrophages that were unstimulated or stimulated with 10 ng/mL of TNF, IFNγ, IFN-β, transforming growth factor-β, or other ligands for 24 hours and then processed for RNA-sequencing^65^. We also generated an RNA-seq dataset mouse bone marrow-derived macrophages left unstimulated or stimulated with 10 ng/mL of TNF, IFNγ, IFN-β, or transforming growth factor-β for 6 hours. For both datasets, counts were normalized by DESeq2’s median of ratios method and biological replicates were averaged to generate average normalized counts for each cytokine condition. The average normalized counts were used to calculate a max to second-max ratio for the 4 cytokine conditions to determine gene specificity. Next, gene expression was compared across all stimulation conditions and filtered to only include genes that were induced relative to the untreated cells by a log_2_ fold change of 1 by IFNγ or IFN-β and to exclude genes induced by a log_2_ fold change of 1.5 by TNF or transforming growth factor-β. Using this filtered gene list, a ratio was calculated of the log_2_ fold change following IFNγ stimulation to the log_2_ fold change following IFN-β stimulation. For the human dataset, IFN-β specific genes were defined as those having a fold change ratio < 0, a log_2_ fold change upon IFN-β stimulation > 1, an average normalized count following IFN-β stimulation > 2000 and a max to second-max ratio > 2.5. Human IFNγ specific genes were defined as those with a fold change ratio > 1.5, a log_2_ fold change upon IFNγ stimulation > 2, an average normalized count following IFNγ stimulation > 1000 and a max to second-max ratio > 2.5. Mouse macrophage IFN-β specific genes were defined as those with a fold change ratio < 0.66, a log_2_ fold change upon IFN-β stimulation > 4, an average normalized count following IFN-β stimulation > 4000 and a max to second-max ratio > 3. Mouse IFNγ specific genes were defined as those that had a fold change ratio > 1.5, a log_2_ fold change upon IFNγ stimulation > 2, an average normalized count following IFNγ stimulation > 500 and a max to second-max ratio > 3.

Human IFN-β and IFNγ gene signatures were validated by examining their induction following human macrophage stimulation with a variety of cytokines, such as IL-4, IL-6 and IL-10, using the published RNA-seq dataset (GEO: GSE20251). The specificity of the mouse macrophage IFNγ signature was validated by stimulating bone marrow-derived macrophages for >16 hours with 10 ng/mL of IFNγ, IFN-β, or nothing and then examining CXCL9 expression by flow cytometric analysis of intracellular staining with PE-labeled anti-mouse CXCL9 antibody (MIG-2F5.5; BioLegend). The mouse gene signatures were then used to score cells in the mouse myeloid scRNA-seq dataset based on their gene expression with the UCell R package^117^.

### QUANTIFICATION AND STATISTICAL ANALYSIS

Group sizes were informed by the results of preliminary experiments and power calculations. The number of animals in each figure is indicated in the legends as n = x mice per group. Statistical significance was determined using Prism (GraphPad) software for unpaired one-tailed or two-tailed Student t test when comparing two populations, one-way or two-way ANOVA tests with Tukey’s or Sidak’s multiple comparisons test when comparing multiple groups. Prism (GraphPad) was also used to calculate linear correlations and R^2^. R was used to calculate statistical significance for bulk RNA- and scRNA-sequencing datasets using the Wald test with multiple testing correction by the Benjamini and Hochberg method and the Wilcoxon Rank-Sum test with Bonferroni correction, respectively. *p < 0.05, **p < 0.01, ***p < 0.001, ****p < 0.0001, n.s. = not significant. See figure legends for more information on statistical tests.

## Supplementary Material

1 **Supplementary Figure 1. Identifying innate immune cell populations in *Mtb*-infected lungs by flow cytometry and scRNA-seq. Related to** Figure 1 and 2. (**A**) Gating strategy for identifying neutrophils, eosinophils, monocytes, DCs, AMs, IMs, conventional type 1 DCs (cDC1), conventional type 2 DCs (cDC2) and *Mtb*-infected cells. Very few *Mtb*^+^ cells were detected among the lymphoid and non-hematopoietic-derived cells. (**B**) Cell clustering by mRNA expression, protein expression, or combined mRNA and protein expression (B6 and *Sp140*^−/−^ combined). (**C**) Cell clustering by biological replicate in naïve and infected B6 and *Sp140*^−/−^ mice. (**D**) Protein and mRNA expression of lineage-defining markers used for cluster classification. Markers used for annotating (**E**) neutrophil and (**F**) monocyte and macrophage clusters based on maturity, activation status, and specific gene expression. The data represents a total of 10 mice (n = 3 for the infected lung samples and n = 2 for the naïve lung samples from B6 and *Sp140*^−/−^ mice).

2 **Supplementary Figure 2. Naïve *Sp140***^**−/−**^ **mice exhibit minimally altered immune cell numbers by flow cytometry and scRNA-seq. Related to** Figure 2. Comparison of innate and adaptive immune cell numbers between B6 (n = 7; closed circles) and *Sp140*^−/−^ (n = 8; open circles) (**A**) spleen, (**B**) lung, (**C**) and thymus. (**D**) Clustering of myeloid cells from naïve lungs of B6 (n = 2) and *Sp140*^−/−^ (n = 2) mice. (**E**) Differentially expressed genes between B6 and *Sp140*^−/−^ AMs, IMs, monocytes, and neutrophils. Greater fold change indicates higher expression in B6 relative to *Sp140*^−/−^. The bars in (A), (B), and (C) represent the median. Pooled data from two independent experiments are shown in (A), (B), and (C). Statistical significance was calculated by multiple unpaired t tests in (A), (B), and (C), and by Wilcoxon Rank-Sum test with Bonferroni correction in (B). *p < 0.05.

3 **Supplementary Figure 3. I-Tomcat cells express TdTomato following *in vitro* stimulation, but the TdTomato signal is undetectable *in vivo* during *Mtb* infection. Using I-Tomcat Ai6 mice, pDC, IMs, and monocytes are the major type I interferon producing cells following *Mtb* infection in B6 and *Sp140***^**−/−**^ **mice. Related to** Figure 3. (**A**) We targeted the mouse *Ifnb* locus immediately downstream of the ORF and upstream of the endogenous polyadenylation (pA) site using CRISPR in C57BL/6 ES cells, inserting a reporter cassette containing the indicated elements. Targeted mice were then bred to FLPer mice to excise the Neo cassette.(**B**) Representative flow cytometry histogram of TdTomato expression by I-Tomcat bone marrow-derived macrophages that were unstimulated (grey) or stimulated with poly I:C (red line). (**C**) Representative flow cytometry plot of TdTomato expression in immune cells in *Mtb*-infected lungs of *Sp140*^−/−^ I-Tomcat^−/−^, *Sp140*^−/−^ I-Tomcat^+/−^, and *Sp140*^−/−^ I-Tomcat^+^/^+^ mice 25 days after infection. (**D**) Representative flow cytometry plot of TdTomato expression in AM, pDCs, monocytes, and IM from *Mtb*-infected lungs of *Sp140*^−/−^ I-Tomcat^+^/^+^ mice 19 days post-infection or (**E**) 25 days post-infection. Frequency of Ai6 expression by immune cell type in the lungs of *Mtb*-infected (**F**) *Sp140*^−/−^ I-Tomcat Ai6 and (**G**) I-Tomcat Ai6 mice. The bars in (E) and (F) represent the median. Lungs were analyzed 25 days after *Mtb* infection. Pooled data from two independent experiments. Statistical significance was calculated by one-way ANOVA with Tukey’s multiple comparison test. *p < 0.05, **p < 0.01, ***p < 0.001, ****p < 0.0001.

4 **Supplementary Figure 4. pDC-DTR mice specifically deplete pDCs without affecting major lung immune cell populations, and CD123 also identifies pDCs in *Mtb*-infected human lymph node samples. Related to** Figure 5. (**A**) Representative flow cytometry plot of splenic pDCs in *Sp140*^+/−^ pDC-DTR mice treated with PBS or DT from days 12 to 24 after *Mtb* infection. (**B**) RNA-sequencing validation of *Sp140* expression and pDC depletion efficiency based on *Siglech* expression in *Sp140*^+/−^ pDC-DTR mice treated with PBS or DT from days 12 to 24 after *Mtb* infection. (**C**) Number of various immune cell populations in *Mtb*-infected lungs of *Sp140*^+/−^ pDC-DTR (n = 13; filled circles) and *Sp140*^+/−^ mice (n = 13; open circles). Mice received DT from days 12 to 24 post-infection. (**D**) anti-CD123 (brown) and hematoxylin staining on *Mtb*-infected human lymph nodes. Mouse lungs and spleens were harvested 25 days after infection. Pooled data from two independent experiments are shown in (B) and (C). The bars in (C) represent the median. Statistical significance in (B) was calculated by the Wald test with multiple testing correction using the Benjamini and Hochberg method and in (C) by multiple unpaired t tests. *p < 0.05, ***p < 0.001, ****p < 0.0001.

5 **Supplementary Figure 5. Generating gene signatures for identifying IFN**γ **and type I IFN responding cells. Related to** Figure 7. (**A**) Plots depicting the log_2_ fold change of genes upregulated in human macrophages or mouse bone marrow-derived macrophages following stimulation with IFNγ or IFN-β. Dots colored red indicate genes used for the IFNγ signaling gene signature while blue dots indicate those used for the gene signature for type I IFN responsiveness. Representative genes induced preferentially by IFNγ or IFN-β are labeled. (**B**) Number of genes upregulated in human macrophages following stimulation with each indicated cytokine and (**C**) Number of type I or II IFN gene signature genes induced after cytokine stimulation. (**D**) Representative flow cytometry histogram of CXCL9 expression by mouse bone marrow-derived macrophages that were untreated (grey), IFN-β stimulated (blue), or IFNγ stimulated (red).

6 **Supplementary Figure 6. Applying gene signatures for identifying IFN**γ **and type I IFN responding cells to the myeloid scRNA-seq dataset. Related to** Figure 7. (**A**) List of genes from the cytokine stimulated mouse macrophages that were used for the gene signature for IFNγ or type I IFN responsiveness. (**B**) IFNγ gene signature or (**C**) type I IFN gene signature expression visualized by wnnUMAP plots of naïve, bystander, or *Mtb*-infected lung myeloid cells from B6 and *Sp140*^−/−^ mice. (**D**) IFNγ and type I IFN gene signature expression on neutrophils, monocytes, IM, and AM from naïve B6 and *Sp140*^−/−^ mouse lungs. The red line indicates the 99% cutoff used to classify cells as type I or II IFN responders. (**E**) Comparison of naïve, bystander, and *Mtb*-infected cells classified as cells that responded to type I IFN (blue), type II IFN (red), both (purple), or neither (grey) in lungs (B6 and *Sp140*^−/−^ combined).

7 **Supplementary Figure 7. IFN**γ **receptor and IFN**γ **responsive genes are expressed at lower levels in *Mtb*-infected IMs in *Sp140***^**−/−**^ **relative to B6 mice. Related to** Figure 7. (**A**) *Ifngr1* and *Ifngr2* expression on bystander and *Mtb*-infected cells from B6 and *Sp140*^−/−^ mice. (**B**) Expression of *Ifngr1* and *Ifngr2*, (**C**) representative type I IFN stimulated genes, including *Isg15*, *Ifit1*, and *Oasl1,* and (**D**) representative type II IFN stimulated genes, including *H2-Ab1* mRNA, MHC II protein, and *Cxcl9* mRNA on bystander and *Mtb*-infected IM and ISG^+^ IM from B6 and *Sp140*^−/−^ mice. (**E**) Gene ontology term enrichment of the Hallmark Interferon-a Response and the REACTOME Translation terms on *Mtb*-infected IM and ISG^+^ IM from B6 and *Sp140*^−/−^ mice. Statistical significance in (B), (C), and (D) was calculated by non-parametric Wilcoxon rank sum test with Bonferroni correction. ***p < 0.001, ****p < 0.0001.

8

## Figures and Tables

**Table:** Key resources table

REAGENT or RESOURCE	SOURCE	IDENTIFIER
Antibodies		
TruStain FcX PLUS (anti-mouse CD16/32) clone S17011E	BioLegend	Cat # 156604; RRID:AB_2783138
BUV496 anti-mouse CD45 clone 30-F11	BD Biosciences	Cat # 749889; RRID:AB_2874129
APC anti-mouse CD64 clone X54–5/7.1	BioLegend	Cat # 139306; RRID:AB_1121939 1
BV480 anti-mouse B220 clone RA3–6B2	BD Biosciences	Cat # 565631; RRID:AB_2739311
BV480 anti-mouse CD90.2 clone 53–2.1	BD Biosciences	Cat # 566082; RRID:AB_2739494
APC-Fire 750 anti-mouse Ly6G clone 1A8	BioLegend	Cat # 127652; RRID:AB_2616733
BUV395 anti-mouse CD11b clone M1/70	BD Biosciences	Cat # 563553; RRID:AB_2738276
BUV737 anti-mouse CD11c clone HL3	BD Biosciences	Cat # 612796; RRID:AB_2870123
APC-R700 anti-mouse Siglec F clone E50–2440	BD Biosciences	Cat # 565183; RRID:AB_2739097
PE anti-mouse MerTK clone DS5MMER	Thermo Fisher Scientific	Cat # 12–5751-82; RRID:AB_2572623
Super Bright 645 anti-mouse MHC II clone M5/114.15.2	Thermo Fisher Scientific	Cat # 64–5321-82; RRID:AB_2662402
BV421 anti-mouse PD-L1 clone MIH5	BD Biosciences	Cat # 564716; RRID:AB_2738911
BV711 anti-mouse Ly6C clone HK1.4	BioLegend	Cat # 128037; RRID:AB_2562630
PE anti-mouse IFNAR-1 clone MAR1–5A3	BioLegend	Cat # 127311; RRID:AB_1134011
PE-Cy7 anti-mouse MerTK clone DS5MMER	Thermo Fisher Scientific	Cat # 25–5751-82; RRID:AB_2573466
APC-eFluor 780 anti-mouse CD11b clone M1/70	Thermo Fisher Scientific	Cat # 47–0112-82; RRID:AB_1603193
BUV395 anti-mouse CCRL2 clone BZ2E3	BD Biosciences	Cat # 743689; RRID:AB_2741676
BUV563 anti-mouse Ly6G clone 1A8	BD Biosciences	Cat # 612921; RRID:AB_2870206
Percp-Cy5.5 anti-mouse B220 clone RA3–6B2	BioLegend	Cat # 103235; RRID:AB_893356
BV421 anti-mouse Siglec H clone 440c	BD Biosciences	Cat # 566581; RRID:AB_2739747
BV480 anti-mouse CD19 clone 1D3	BD Biosciences	Cat # 566167; RRID:AB_2739564
BV605 anti-mouse MHC II clone M5/114.15.2	BD Biosciences	Cat # 563413; RRID:AB_2738190
BV785 anti-mouse Ly6C clone HK1.4	BioLegend	Cat # 128041; RRID:AB_2565852
BV605 anti-mouse CD4 clone GK1.5	BioLegend	Cat # 100451; RRID:AB_2564591
BUV805 anti-mouse CD8ɑ clone 53–6.7	BD Biosciences	Cat # 612898; RRID:AB_2870186
PE-Cy7 anti-mouse PDCA-1 clone eBio927	Thermo Fisher Scientific	Cat # 25–3172-80; RRID:AB_2573439
PE-Cy7 anti-mouse CD63 clone NVG-2	BioLegend	Cat # 143909
Percp-eFluor 710 anti-mouse iNOS clone CXNFT	Thermo Fisher Scientific	Cat # 46–5920-82; RRID:AB_2688059
BV785 anti-mouse CD206 clone C068C2	BioLegend	Cat # 141729; RRID:AB_2565823
Anti-mouse BST2 clone 927	BioXCell	Cat # BE0311; RRID:AB_2736991
Rat IgG2b isotype antibody clone LTF-2	BioXCell	Cat # BE0090; RRID:AB_1107780
Anti-mouse Ly6G clone 1A8	BioXCell	Cat # BE0075–1; RRID:AB_1107721
Rat IgG2a isotype antibody clone 2A3	BioXCell	Cat # BE0089; RRID:AB_1107769
Anti-human CD123 clone 6h6	Thermo Fisher Scientific	Cat # 14–1239-82; RRID:AB_467453
Anti-human CD303 clone 124B3.13	Dendritics	Cat # DDX0043; RRID:AB_1149764
BV421 anti-mouse SIRP𝘢 clone P84	BD Biosciences	Cat # 740071; RRID:AB_2739835
Pacific Blue anti-mouse B220 clone RA3–6B2	BioLegend	Cat # 103227; RRID:AB_492876
eF506 anti-mouse CD4 clone RM4–5	Thermo Fisher Scientific	Cat # 69–0042-80; RRID:AB_2637458
AF647 anti-mouse Ly6G clone 1A8	BioLegend	Cat # 127609; RRID:AB_1134162
BV421 anti-mouse Ly6G clone 1A8	BioLegend	Cat # 127628; RRID:AB_2562567
Rabbit polyclonal anti-citrullinated histone-H3 (citrulline R2, R8, R17)	Abcam	Cat # ab5103; RRID:AB_304752
AF488 donkey anti-rabbit secondary clone Poly4064	BioLegend	Cat # 406416; RRID:AB_2563203
APC anti-mouse Ly6G clone 1A8	BioLegend	Cat # 127614; RRID:AB_2227348
TotalSeq-A anti-mouse Ly6G clone 1A8	BioLegend	Cat # 127655; RRID:AB_2749962
TotalSeq-A anti-mouse Ly6C clone HK1.4	BioLegend	Cat # 128047; RRID:AB_2749961
TotalSeq-A anti-mouse CD44 clone IM7	BioLegend	Cat # 103045; RRID:AB_2734154
TotalSeq-A anti-mouse CD169 clone 3D6.112	BioLegend	Cat # 142425; RRID:AB_2783106
TotalSeq-A anti-mouse CD274 clone MIH6	BioLegend	Cat # 153604; RRID:AB_2783125
TotalSeq-A anti-mouse Siglec F clone S17007L	BioLegend	Cat # 155513; RRID:AB_2832540
TotalSeq-A anti-mouse CSF1R clone AFS98	BioLegend	Cat # 135533; RRID:AB_2734198
TotalSeq-A anti-mouse CD11b clone M1/70	BioLegend	Cat # 101265; RRID:AB_2734152
TotalSeq-A anti-mouse CD86 clone GL-1	BioLegend	Cat # 105047; RRID:AB_2750348
TotalSeq-A anti-mouse MHC II clone M5/114.15.2	BioLegend	Cat # 107653; RRID:AB_2750505
TotalSeq-A anti-mouse CX3CR1 clone SA011F11	BioLegend	Cat # 149041; RRID:AB_2783121
TotalSeq-A anti-mouse Hashtag 1	BioLegend	Cat # 155801; RRID:AB_2750032
TotalSeq-A anti-mouse Hashtag 2	BioLegend	Cat # 155803; RRID:AB_2750033
TotalSeq-A anti-mouse Hashtag 3	BioLegend	Cat # 155805; RRID:AB_2750034
TotalSeq-A anti-mouse Hashtag 4	BioLegend	Cat # 155807; RRID:AB_2750035
TotalSeq-A anti-mouse Hashtag 5	BioLegend	Cat # 155809; RRID:AB_2750036
TotalSeq-A anti-mouse Hashtag 6	BioLegend	Cat # 155811; RRID:AB_2750037
PE anti-mouse B220 clone RA3–6B2	Tonbo Biosciences	Cat # 50–0452-U100; RRID:AB_2621764
PE anti-mouse CD90.2 clone 30-H12	Tonbo Biosciences	Cat # 50–0903-U025; RRID:AB_2940772
BV785 anti-mouse CD45.2 clone 104	BioLegend	Cat # 109839; RRID:AB_2562604
Pacific Blue anti-mouse B220 clone RA3–6B2	BioLegend	Cat # 103230; RRID:AB_492877
Pacific Blue anti-mouse CD90.2 clone 53–2.1	BioLegend	Cat # 140305; RRID:AB_1064533 5
PE anti-mouse F4/80 clone BM8	Thermo Fisher Scientific	Cat # 12–4801-80; RRID:AB_465922
PE anti-mouse CXCL9 clone MIG-2F5.5	BioLegend	Cat # 515603; RRID:AB_2245490
Bacterial and virus strains		
Bacteria: *Mtb* strain Erdman	A gift from Sarah Stanley, University of California, Berkeley	N/A
Bacteria: *Mtb*-Wasabi	This study	N/A
Bacteria: *Mtb*-mCherry	This study	N/A
Biological samples		
Human lung and lymph node samples	Surgical pathology archives of Emory University Hospital	N/A
Chemicals, peptides, and recombinant proteins		
Ghost Dye Violet 510	Tonbo Biosciences	Cat # 13–0870-T500
Super Bright Complete Staining Buffer	Thermo Fisher Scientific	Cat # SB-4401–75
True-Stain Monocyte Blocker	BioLegend	Cat # 426102
Cytofix/cytoperm	BD biosciences	Cat # 554722
AccuCheck Counting Beads	Invitrogen	Cat # PCB100
Vector Laboratories Hematoxylin and Eosin Stain Kit	Thermo Fisher Scientific	Cat # NC1470670
Diphtheria toxin	Millipore Sigma	Cat # D0564–1MG
DreamTaq Green PCR Master Mix (2X)	Thermo Fisher Scientific	Cat # K1082
Middlebrook 7H9 Broth (Dehydrated)	Thermo Fisher Scientific	Cat # R454012
BBL seven H11 agar base	BD biosciences	Cat # 212203
Middlebrook OADC	Thermo Fisher Scientific	Cat # b12351
Hygromycin B Gold	Invivogen	Cat # ant-hg-1
Kanamycin	Millipore Sigma	Cat # K4000–5G
Liberase TM	Roche	Cat # 5401127001
Dnase I	Roche	Cat # 11284932001
Newborn Calf Serum	Thermo Fisher Scientific	Cat # 26010074
Trizol LS	Thermo Fisher Scientific	Cat # 10296010
M-CSF	Vance lab	N/A
IFN-β	BioLegend	Cat # 581302
IFNg	Peprotech	Cat # 315–05
Tumor necrosis factor	Peprotech	Cat # 315–01A
Transforming growth factor-β	BioLegend	Cat # 763102
TRK lysis buffer	Omega Bio-Tek	Cat # PR021
2-mercaptoethanol	Thermo Fisher Scientific	Cat # 21985023
Ultracomp eBeads Plus	Thermo Fisher Scientific	Cat # 01–3333-42
Sytox Blue Dead Cell Stain	Invitrogen	Cat # S11348
RNaseOUT Recombinant Ribonuclease Inhibitor	Invitrogen	Cat # 10777019
Critical commercial assays		
RNeasy Micro	Qiagen	Cat # 74004
E.Z.N.A Total RNA Kit I	Omega Bio-Tek	Cat # R6834–02
EasySep APC Positive Selection Kit II	StemCell Technologies	Cat # 17681
Chromium Single Cell 3’ Reagent Kit v3.1 chemistry	10X Genomics	Cat # 1000268
Deposited data		
scRNA-seq of *Mtb*-infected non-human primates	Esaulova et al.^51^	GEO: GSE149758
Bulk RNA-seq of cytokine stimulated human macrophages	Nilsson et al.^65^	GEO: GSE20251
Bulk RNA-seq of cytokine stimulated mouse bone marrow-derived macrophages	This study	GEO: GSE232827
Bulk RNA-seq of *Mtb*-infected lungs from pDC depleted *Sp140*^+/−^ and *Sp140*^−/−^ mice	This study	GEO: GSE232922
scRNA-seq of myeloid cells from naïve and *Mtb*-infected B6 and *Sp140*^−/−^ mice	This study	GEO: GSE216023
Experimental models: Organisms/strains		
Mouse: C57BL/6J	The Jackson Laboratory	Cat # 000664; RRID:IMSR_JAX:0 00664
Mouse: *Ifnar1*^−/−^: B6.129S2-Ifnar1^tm1Agt^/Mmjax	The Jackson Laboratory	Cat # 032045-JAX RRID:MMRRC_03 2045-JAX
Mouse: Ai6: B6.Cg-Gt(ROSA)26Sor^tm6(CAG-ZsGreen1)Hze^/J	The Jackson Laboratory	Cat # 007906 RRID:IMSR_JAX:0 07906
Mouse: *Ifnar1*^fl^: B6(Cg)-Ifnar1^tm1.1Ees^/J	The Jackson Laboratory	Cat # 028256 RRID:IMSR_JAX:0 28256
Mouse: Mrp8^Cre^: B6.Cg-Tg(S100A8-cre,-EGFP)1Ilw/J	The Jackson Laboratory	Cat # 021614 RRID:IMSR_JAX:0 21614
Mouse: LysM^Cre^: B6.129P2-Lyz2^tm1(cre)Ifo^/J	The Jackson Laboratory	Cat # 004781 RRID:IMSR_JAX:0 04781
Mouse: C57BL/6J-*Ticam1^Lps2^*/J (*Ticam1*^−/−^)	The Jackson Laboratory	Cat # 005037 RRID:IMSR_JAX:0 05037
Mouse: C57BL/6N-*Unc93b1^tm1(KOMP)Vlcg^*/Mmucd (*Unc93b1*^−/−^)	MMRRC, KOMP repository, Regeneron Pharmaceuticals	Cat # 050296-UCD RRID:MMRRC_05 0296-UCD
Mouse: I-Tomcat: *Ifnb1*-Tomato-Cre-pA Terminator	This study	N/A
Mouse: FLPer: B6N.129S4-*Gt(ROSA)26Sor^tm1(FLP1)Dym^*/J	The Jackson Laboratory	Cat # 016226 RRID:IMSR_JAX:0 16226
Mouse: CD64^Cre^: B6-Fcgr1^tm2Ciphe^	Scott et al.^106^	N/A
Mouse: pDC-DTR: B6-Tg(CLEC4C-HBEGF)956Cln/J	The Jackson Laboratory	Cat # 014176; RRID:IMSR_JAX:0 14176
Mouse: *Sp140*^−/−^	Ji et al.^31^	N/A
Recombinant DNA		
pTEC15	Takaki et al.^36^	Addgene plasmid # 30174
pMSP12::mCherry	A gift from Lalita Ramakrishnan, University of Cambridge	Addgene plasmid # 30167
Software and algorithms		
Chrysalis	Kotov et al.^55^	https://github.com/Histo-cytometry/Chrysalis
Generate Compensation Matrix	Kotov et al. ^55^	https://github.com/Histo-cytometry/Chrysalis
Imaris version 9.9.1	Bitplane	N/A
FlowJo version 10	BD Biosciences	N/A
Trimmomatic v.0.36	Bolger et al.^109^	https://github.com/usadellab/Trimmomatic
STAR aligner v.2.5.2b	Dobin et al.^110^	https://github.com/alexdobin/STAR
CellRanger version 4.0.0	10X Genomics	N/A
CITE-Seq-Count v.1.4.3	Roelli et al.^113^	https://github.com/Hoohm/CITE-seq-Count
R version 3.16	R Development Core Team^114^	http://www.r-project.org/
RStudio “Cherry Blossom” Release	Posit	https://posit.co/products/open-source/rstudio/
DESeq2 v.1.38.3	Love et al.^111^	https://bioconductor.org/packages/release/bioc/html/DESeq2.html
Seurat v.4.1.1	Hao et al.^42^	https://satijalab.org/seurat/index.html
EnhancedVolcano v1.16.0	Blighe et al.^116^	http://bioconductor.org/packages/release/bioc/html/EnhancedVolcano.html
Tidyverse v2.0.0	Wickham et al.^115^	https://www.tidyverse.org/
UCell v2.2.0	Andreatta et al.^117^	http://www.bioconductor.org/packages/release/bioc/html/UCell.html
Adobe Illustrator	Adobe.com	N/A
Prism	GraphPad	N/A
Other		
4 laser SH-800 cell sorter	Sony	N/A
LSM 880 laser scanning confocal microscope	Zeiss	N/A
5 laser LSRFortessa analyzer	BD Biosciences	N/A
5 laser Aurora analyzer	Cytek	N/A
GentleMACS	Miltenyi Biotec	N/A
Chromium controller	10X Genomics	N/A

## Highlights

- *Sp140*-deficient mice model type I interferon-driven tuberculosis disease seen in humans
- Plasmacytoid dendritic cells and interstitial macrophages produce IFN during tuberculosis
- Plasmacytoid dendritic cell depletion rescues tuberculosis susceptibility
- Type I interferons act on infected macrophages to impair bacterial control
