# Supplementary material for: Early cellular mechanisms of type I interferon-driven susceptibility to tuberculosis
Source: Cell. Author manuscript; Available in PMC 2023 Dec 30. (PMC10757650; doi:10.1016/j.cell.2023.11.002)
Supplement: 1 — Supplementary Figure 1. Identifying innate immune cell populations in Mtb-infected lungs by flow cytometry and scRNA-seq. Related to Figure 1 and 2. (A) Gating strategy for identifying neutrophils, eosinophils, monocytes, DCs, AMs, IMs, conventional type 1 DCs (cDC1), conventional type 2 DCs (cDC2) and Mtb-infected cells. Very few Mtb+ cells were detected among the lymphoid and non-hematopoietic-derived cells. (B) Cell clustering by mRNA expression, protein expression, or combined mRNA and protein expression (B6 and Sp140−/− combined). (C) Cell clustering by biological replicate in naïve and infected B6 and Sp140−/− mice. (D) Protein and mRNA expression of lineage-defining markers used for cluster classification. Markers used for annotating (E) neutrophil and (F) monocyte and macrophage clusters based on maturity, activation status, and specific gene expression. The data represents a total of 10 mice (n = 3 for the infected lung samples and n = 2 for the naïve lung samples from B6 and Sp140−/− mice). [file NIHMS1947235-supplement-1.pdf]

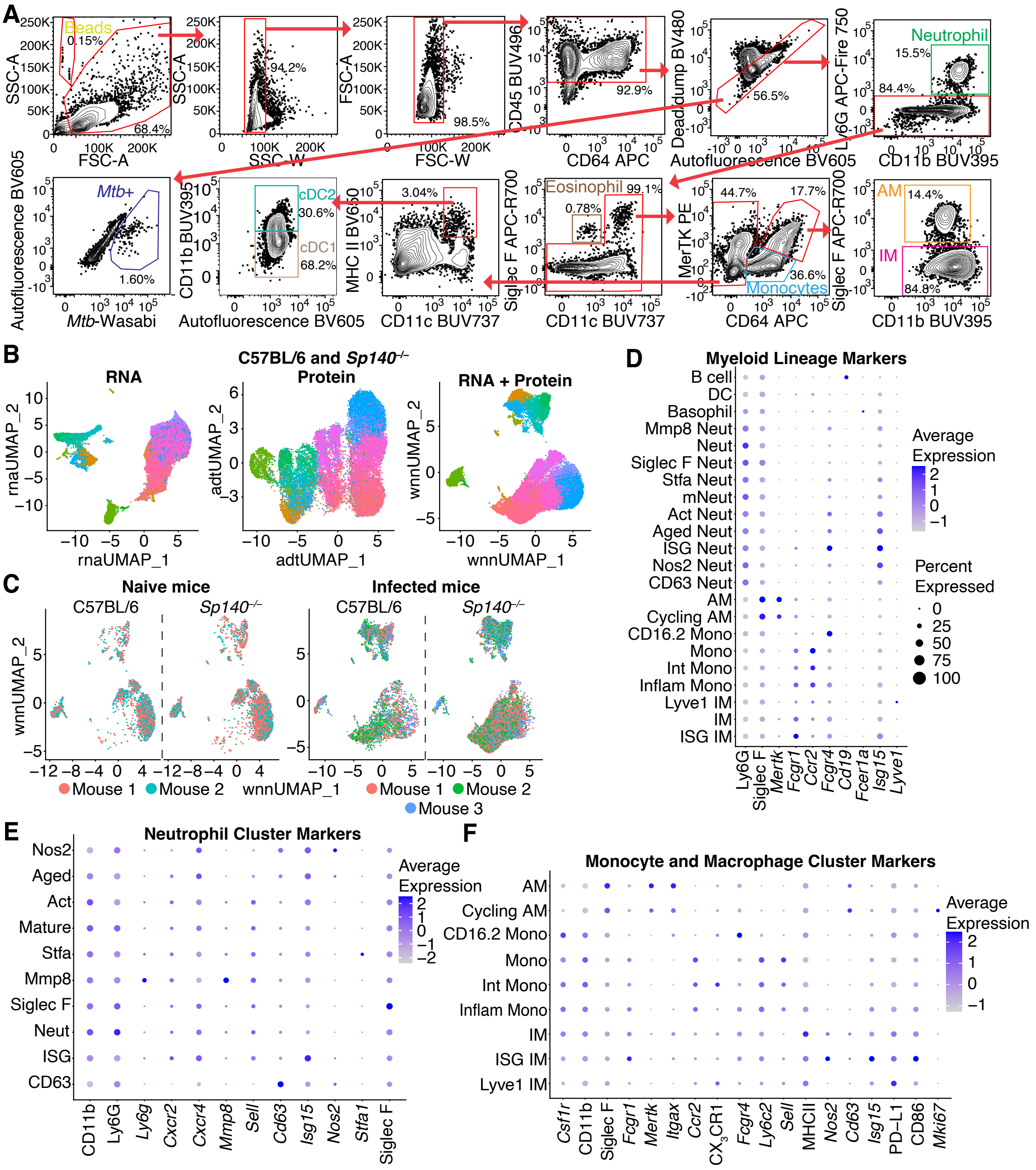

Supplementary Figure 1. Identifying innate immune cell populations in *Mtb*-infected lungs by flow cytomery and scRNA-seq. Related to Figure 1 and 2.
