# Supplementary material for: Early cellular mechanisms of type I interferon-driven susceptibility to tuberculosis
Source: Cell. Author manuscript; Available in PMC 2023 Dec 30. (PMC10757650; doi:10.1016/j.cell.2023.11.002)
Supplement: 2 — Supplementary Figure 2. Naïve Sp140−/− mice exhibit minimally altered immune cell numbers by flow cytometry and scRNA-seq. Related to Figure 2. Comparison of innate and adaptive immune cell numbers between B6 (n = 7; closed circles) and Sp140−/− (n = 8; open circles) (A) spleen, (B) lung, (C) and thymus. (D) Clustering of myeloid cells from naïve lungs of B6 (n = 2) and Sp140−/− (n = 2) mice. (E) Differentially expressed genes between B6 and Sp140−/− AMs, IMs, monocytes, and neutrophils. Greater fold change indicates higher expression in B6 relative to Sp140−/−. The bars in (A), (B), and (C) represent the median. Pooled data from two independent experiments are shown in (A), (B), and (C). Statistical significance was calculated by multiple unpaired t tests in (A), (B), and (C), and by Wilcoxon Rank-Sum test with Bonferroni correction in (B). *p < 0.05. [file NIHMS1947235-supplement-2.pdf]

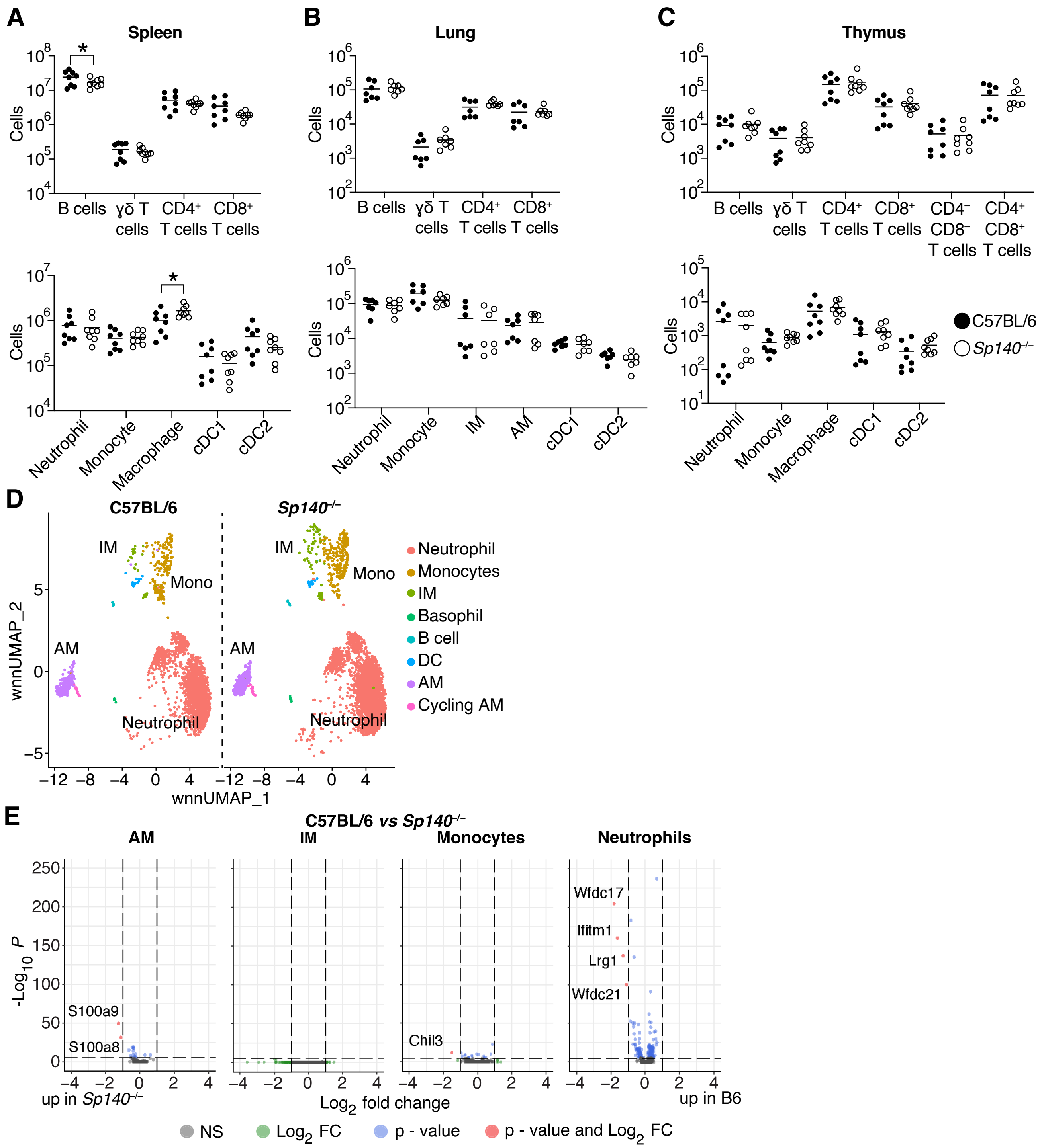

Supplementary Figure 2. Naïve *Sp140*–/– mice exhibit minimally altered immune cell numbers by flow cytometry and scRNA-seq. Related to Figure 2.
