# Supplementary material for: Early cellular mechanisms of type I interferon-driven susceptibility to tuberculosis
Source: Cell. Author manuscript; Available in PMC 2023 Dec 30. (PMC10757650; doi:10.1016/j.cell.2023.11.002)
Supplement: 3 — Supplementary Figure 3. I-Tomcat cells express TdTomato following in vitro stimulation, but the TdTomato signal is undetectable in vivo during Mtb infection. Using I-Tomcat Ai6 mice, pDC, IMs, and monocytes are the major type I interferon producing cells following Mtb infection in B6 and Sp140−/− mice. Related to Figure 3. (A) We targeted the mouse Ifnb locus immediately downstream of the ORF and upstream of the endogenous polyadenylation (pA) site using CRISPR in C57BL/6 ES cells, inserting a reporter cassette containing the indicated elements. Targeted mice were then bred to FLPer mice to excise the Neo cassette.(B) Representative flow cytometry histogram of TdTomato expression by I-Tomcat bone marrow-derived macrophages that were unstimulated (grey) or stimulated with poly I:C (red line). (C) Representative flow cytometry plot of TdTomato expression in immune cells in Mtb-infected lungs of Sp140−/− I-Tomcat−/−, Sp140−/− I-Tomcat+/−, and Sp140−/− I-Tomcat+/+ mice 25 days after infection. (D) Representative flow cytometry plot of TdTomato expression in AM, pDCs, monocytes, and IM from Mtb-infected lungs of Sp140−/− I-Tomcat+/+ mice 19 days post-infection or (E) 25 days post-infection. Frequency of Ai6 expression by immune cell type in the lungs of Mtb-infected (F) Sp140−/− I-Tomcat Ai6 and (G) I-Tomcat Ai6 mice. The bars in (E) and (F) represent the median. Lungs were analyzed 25 days after Mtb infection. Pooled data from two independent experiments. Statistical significance was calculated by one-way ANOVA with Tukey’s multiple comparison test. *p < 0.05, **p < 0.01, ***p < 0.001, ****p < 0.0001. [file NIHMS1947235-supplement-3.pdf]

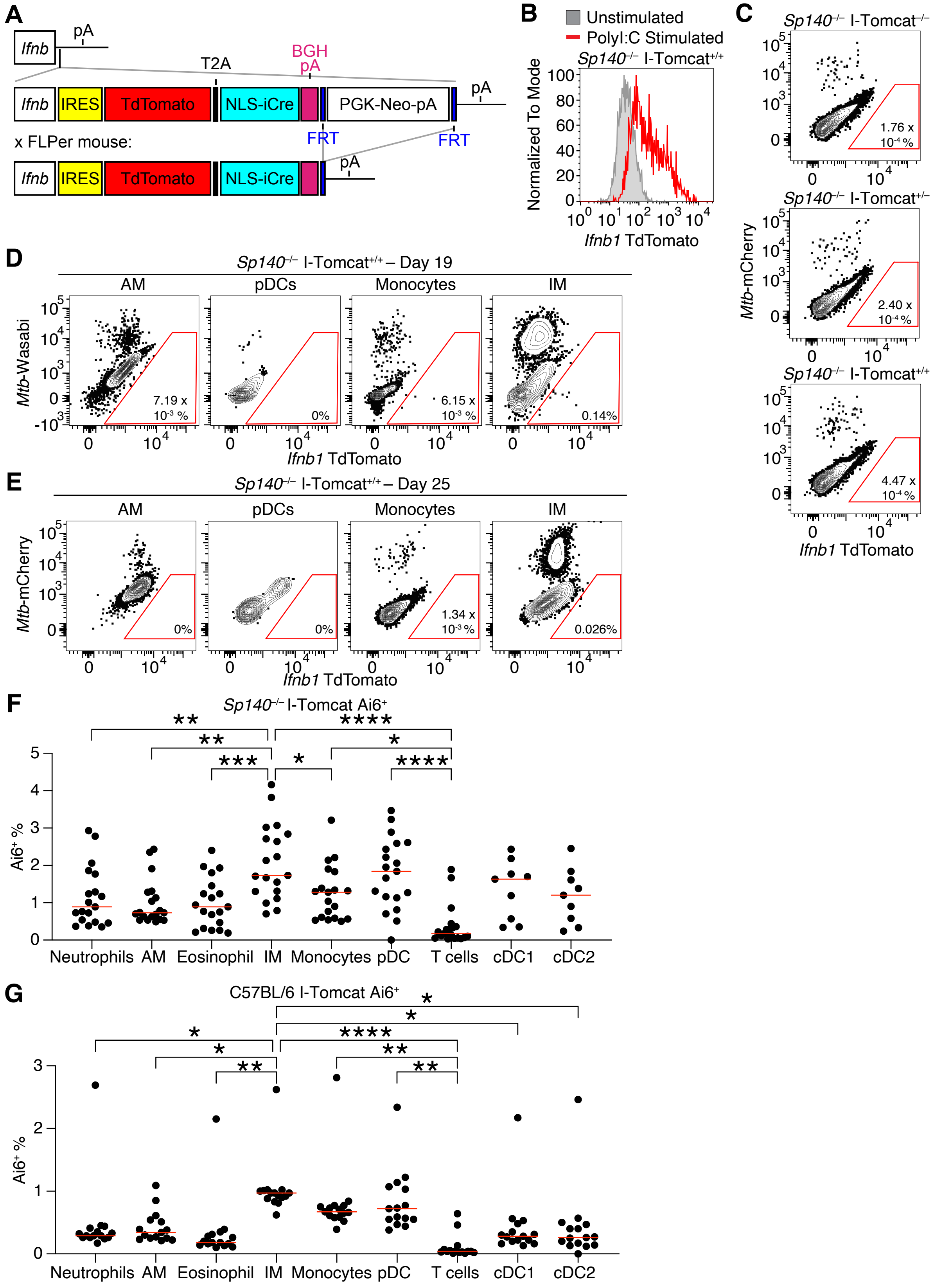

Supplementary Figure 3. I-Tomcat cells express TdTomato following *in vitro* stimulation, but the TdTomato signal is undetectable *in vivo* during *Mtb* infection. Using I-Tomcat Ai6 mice, pDC, IMs, and monocytes are the major type I interferon producing cells following *Mtb* infection in B6 and *Sp140*$^{-/-}$ mice. Related to Figure 3.
