# Supplementary material for: Early cellular mechanisms of type I interferon-driven susceptibility to tuberculosis
Source: Cell. Author manuscript; Available in PMC 2023 Dec 30. (PMC10757650; doi:10.1016/j.cell.2023.11.002)
Supplement: 4 — Supplementary Figure 4. pDC-DTR mice specifically deplete pDCs without affecting major lung immune cell populations, and CD123 also identifies pDCs in Mtb-infected human lymph node samples. Related to Figure 5. (A) Representative flow cytometry plot of splenic pDCs in Sp140+/− pDC-DTR mice treated with PBS or DT from days 12 to 24 after Mtb infection. (B) RNA-sequencing validation of Sp140 expression and pDC depletion efficiency based on Siglech expression in Sp140+/− pDC-DTR mice treated with PBS or DT from days 12 to 24 after Mtb infection. (C) Number of various immune cell populations in Mtb-infected lungs of Sp140+/− pDC-DTR (n = 13; filled circles) and Sp140+/− mice (n = 13; open circles). Mice received DT from days 12 to 24 post-infection. (D) anti-CD123 (brown) and hematoxylin staining on Mtb-infected human lymph nodes. Mouse lungs and spleens were harvested 25 days after infection. Pooled data from two independent experiments are shown in (B) and (C). The bars in (C) represent the median. Statistical significance in (B) was calculated by the Wald test with multiple testing correction using the Benjamini and Hochberg method and in (C) by multiple unpaired t tests. *p < 0.05, ***p < 0.001, ****p < 0.0001. [file NIHMS1947235-supplement-4.pdf]

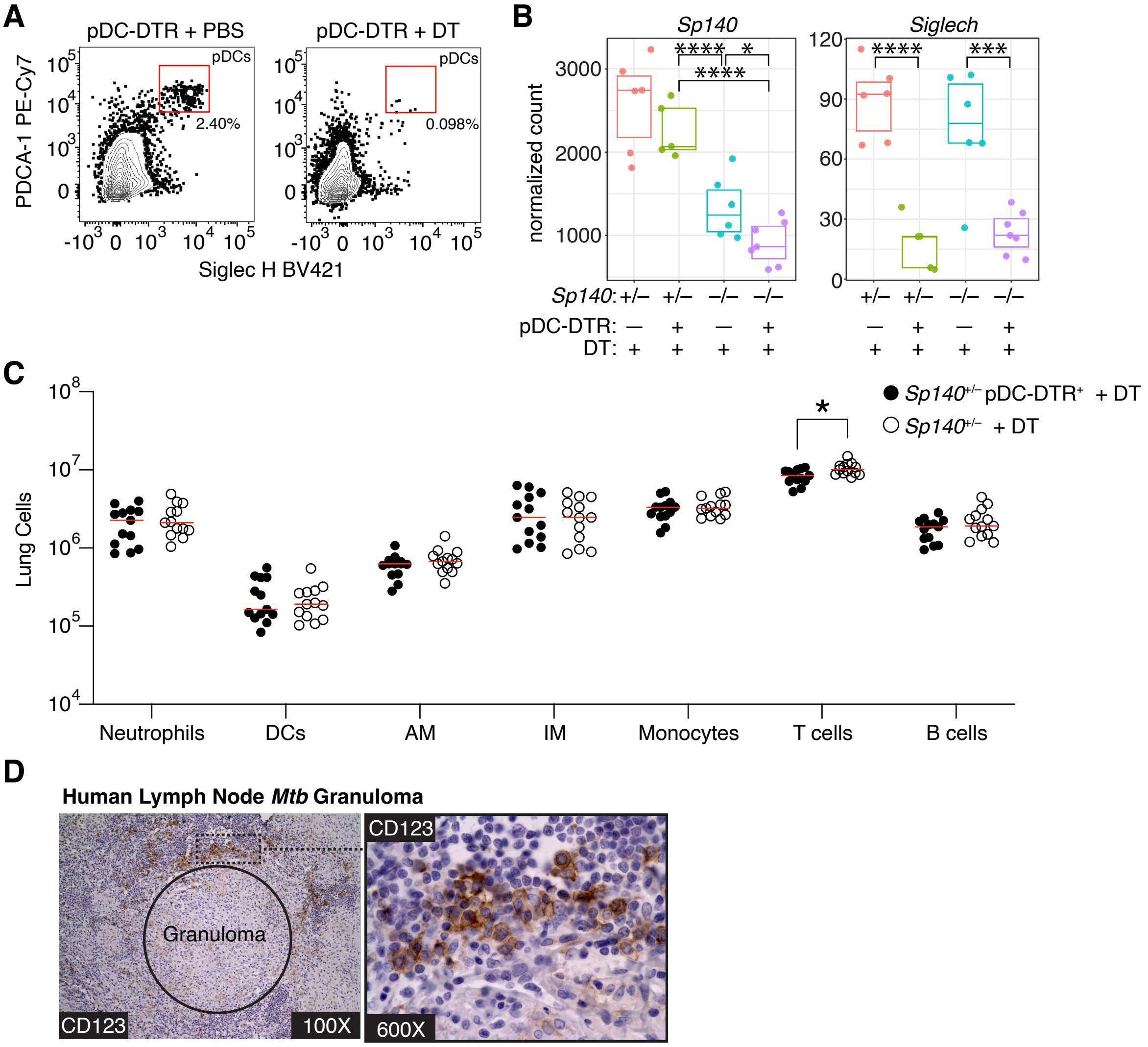

Supplementary Figure 4. pDC-DTR mice specifically deplete pDCs without affecting major lung immune cell populations, and CD123 also identifies pDCs in *Mtb*-infected human lymph node samples. Related to Figure 5.
