# Supplementary material for: Early cellular mechanisms of type I interferon-driven susceptibility to tuberculosis
Source: Cell. Author manuscript; Available in PMC 2023 Dec 30. (PMC10757650; doi:10.1016/j.cell.2023.11.002)
Supplement: 5 — Supplementary Figure 5. Generating gene signatures for identifying IFNγ and type I IFN responding cells. Related to Figure 7. (A) Plots depicting the log2 fold change of genes upregulated in human macrophages or mouse bone marrow-derived macrophages following stimulation with IFNγ or IFN-β. Dots colored red indicate genes used for the IFNγ signaling gene signature while blue dots indicate those used for the gene signature for type I IFN responsiveness. Representative genes induced preferentially by IFNγ or IFN-β are labeled. (B) Number of genes upregulated in human macrophages following stimulation with each indicated cytokine and (C) Number of type I or II IFN gene signature genes induced after cytokine stimulation. (D) Representative flow cytometry histogram of CXCL9 expression by mouse bone marrow-derived macrophages that were untreated (grey), IFN-β stimulated (blue), or IFNγ stimulated (red). [file NIHMS1947235-supplement-5.pdf]

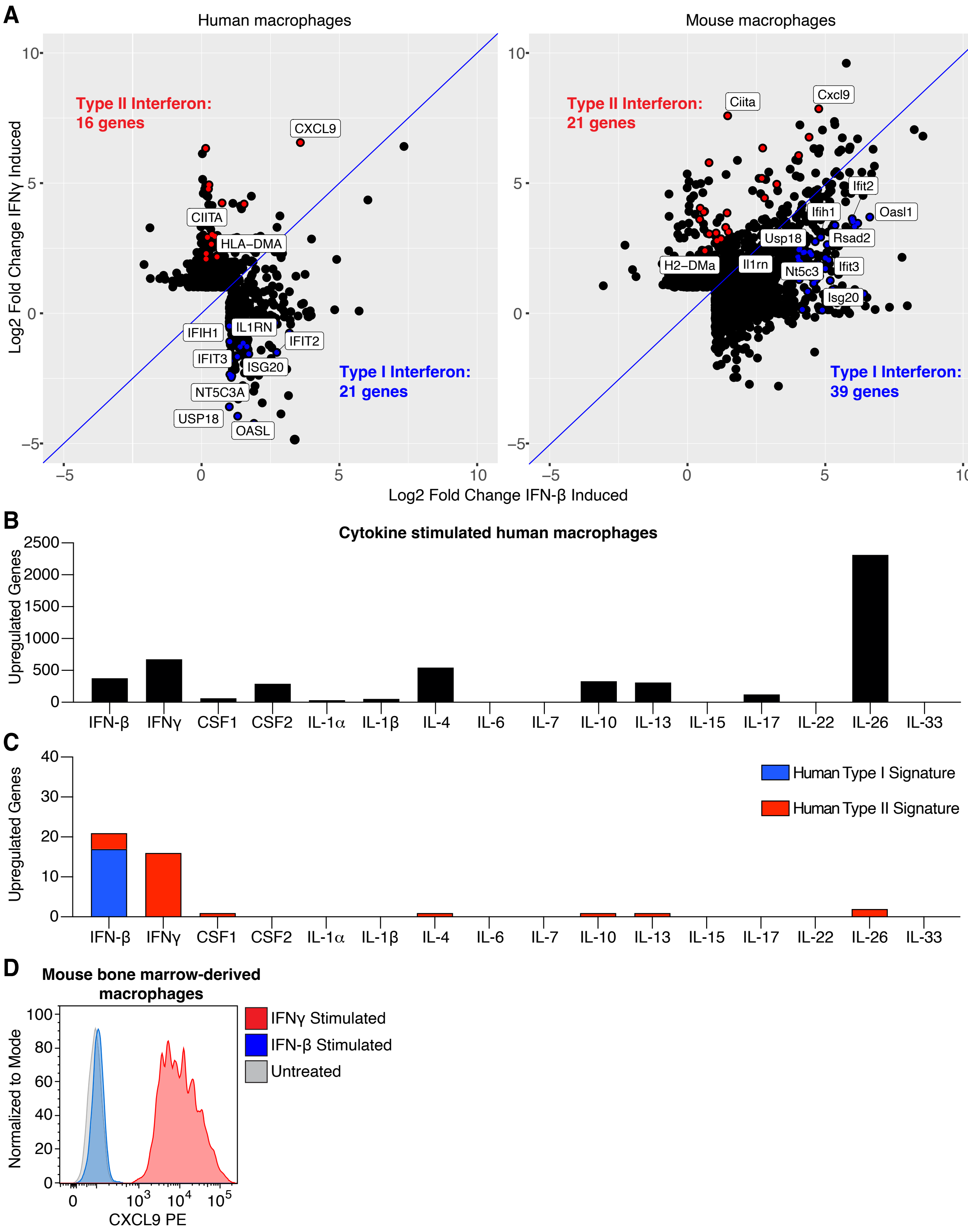

Supplementary Figure 5. Generating gene signatures for identifying IFNγ and type I IFN responding cells. Related to Figure 7.
