# Supplementary material for: Early cellular mechanisms of type I interferon-driven susceptibility to tuberculosis
Source: Cell. Author manuscript; Available in PMC 2023 Dec 30. (PMC10757650; doi:10.1016/j.cell.2023.11.002)
Supplement: 6 — Supplementary Figure 6. Applying gene signatures for identifying IFNγ and type I IFN responding cells to the myeloid scRNA-seq dataset. Related to Figure 7. (A) List of genes from the cytokine stimulated mouse macrophages that were used for the gene signature for IFNγ or type I IFN responsiveness. (B) IFNγ gene signature or (C) type I IFN gene signature expression visualized by wnnUMAP plots of naïve, bystander, or Mtb-infected lung myeloid cells from B6 and Sp140−/− mice. (D) IFNγ and type I IFN gene signature expression on neutrophils, monocytes, IM, and AM from naïve B6 and Sp140−/− mouse lungs. The red line indicates the 99% cutoff used to classify cells as type I or II IFN responders. (E) Comparison of naïve, bystander, and Mtb-infected cells classified as cells that responded to type I IFN (blue), type II IFN (red), both (purple), or neither (grey) in lungs (B6 and Sp140−/− combined). [file NIHMS1947235-supplement-6.pdf]

A

IFN-β Signature Genes	
Ifit1bl1	Ifi213
Ms4a4c	Phf11d
Mmp13	Phf11b
Kdr	Ifit2
Isg20	Herc6
Gadd45g	Mov10
Ddx60	Itpr1
Ifit3b	Oasl2
Tlr3	Abcg1
Slc28a2	Ifi206
Ifi44	Ifih1
Ifit3	Usp18
Ifi209	Mndal
Oasl1	Il1rn
Irf7	Nt5c3
Thbs1	Sp140
Rsad2	Slfn5
Ifi208	
Cmpk2	
Ifit1	

IFNγ Signature Genes
Ciita
Cd74
Socs1
Rnase6
Tagap
Cxcl9
Irf1
H2-Ab1
Hcar2
Irf8
Batf2
Arhgef3
Prr5l
H2-DMa
Cdkn1a
Lrrc8c
Upp1
Naaa
Cd83
Gbp8

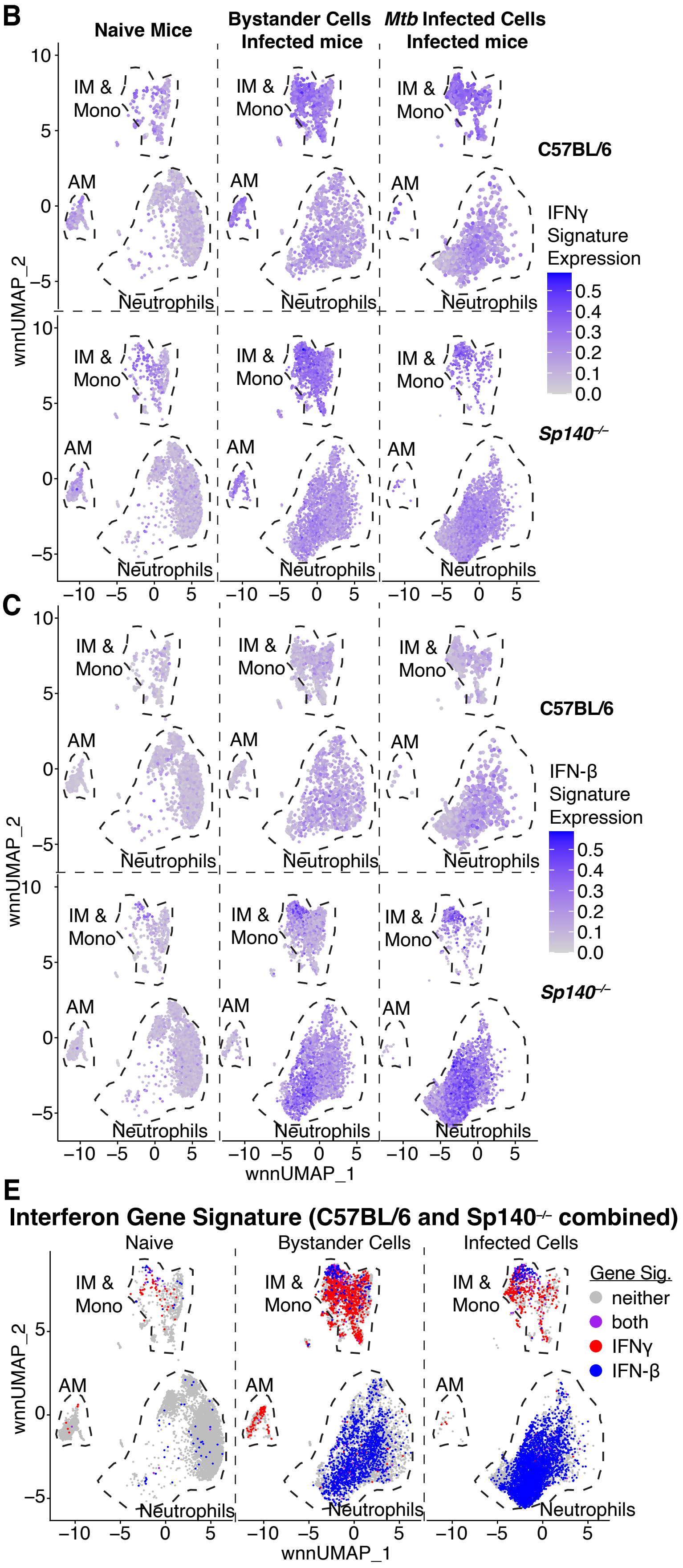

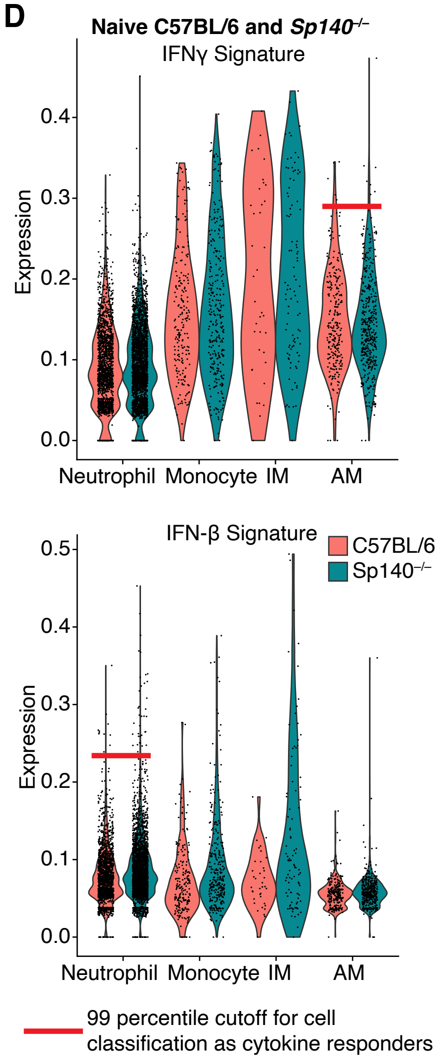

Supplementary Figure 6. Applying gene signatures for identifying IFNγ and type I IFN responding cells to the myeloid scRNA-seq dataset. Related to Figure 7.
