# Supplementary material for: Early cellular mechanisms of type I interferon-driven susceptibility to tuberculosis
Source: Cell. Author manuscript; Available in PMC 2023 Dec 30. (PMC10757650; doi:10.1016/j.cell.2023.11.002)
Supplement: 7 — Supplementary Figure 7. IFNγ receptor and IFNγ responsive genes are expressed at lower levels in Mtb-infected IMs in Sp140−/− relative to B6 mice. Related to Figure 7. (A) Ifngr1 and Ifngr2 expression on bystander and Mtb-infected cells from B6 and Sp140−/− mice. (B) Expression of Ifngr1 and Ifngr2, (C) representative type I IFN stimulated genes, including Isg15, Ifit1, and Oasl1, and (D) representative type II IFN stimulated genes, including H2-Ab1 mRNA, MHC II protein, and Cxcl9 mRNA on bystander and Mtb-infected IM and ISG+ IM from B6 and Sp140−/− mice. (E) Gene ontology term enrichment of the Hallmark Interferon-a Response and the REACTOME Translation terms on Mtb-infected IM and ISG+ IM from B6 and Sp140−/− mice. Statistical significance in (B), (C), and (D) was calculated by non-parametric Wilcoxon rank sum test with Bonferroni correction. ***p < 0.001, ****p < 0.0001. [file NIHMS1947235-supplement-7.pdf]

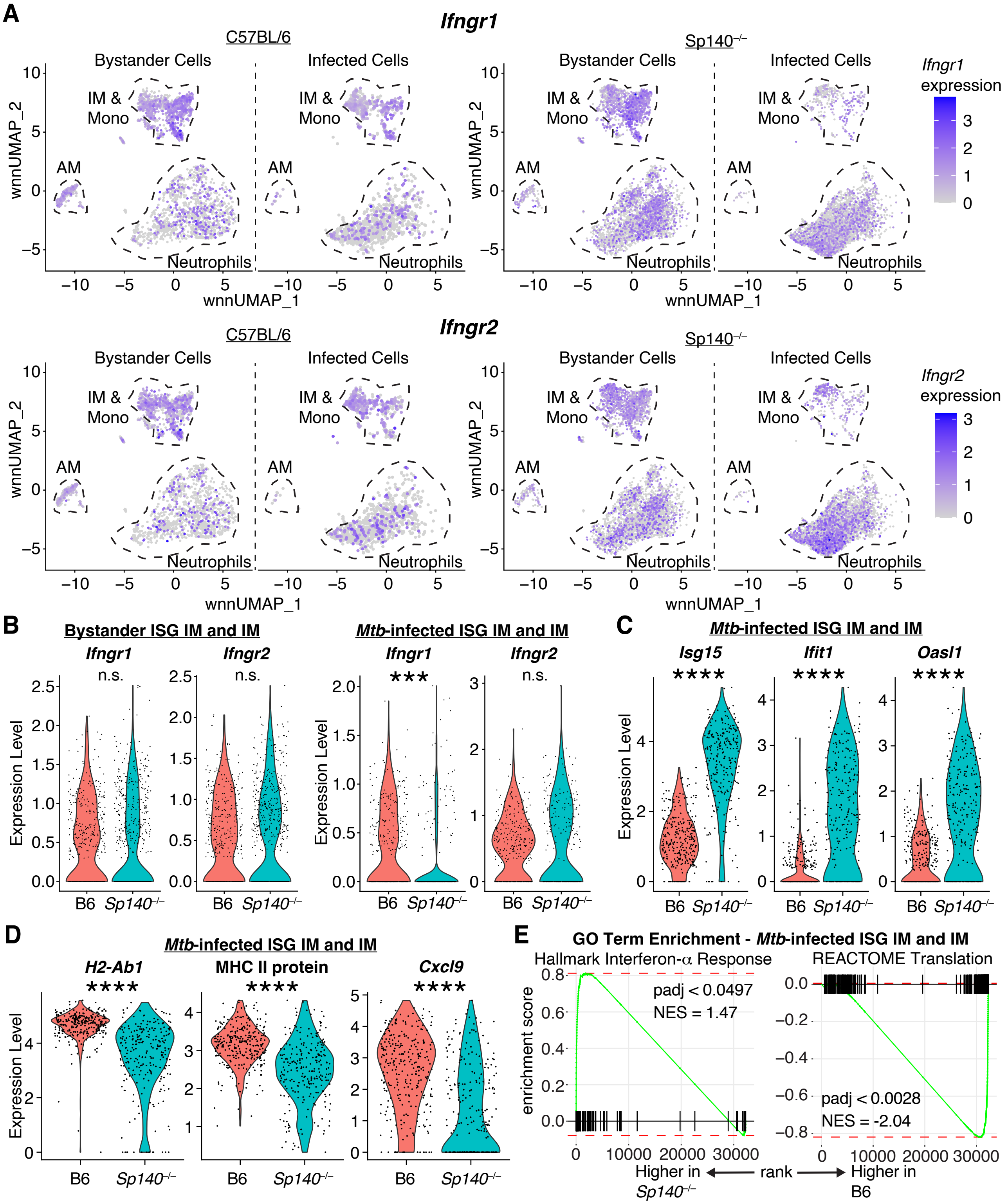

Supplementary Figure 7. IFNγ receptor and IFNγ responsive genes are expressed at lower levels in *Mtb*-infected IMs in *Sp140*$^{-/-}$ relative to B6 mice. Related to Figure 7.
