# Supplementary material for: Early cellular mechanisms of type I interferon-driven susceptibility to tuberculosis
Source: Cell. Author manuscript; Available in PMC 2023 Dec 30. (PMC10757650; doi:10.1016/j.cell.2023.11.002)
Supplement: 8 [file NIHMS1947235-supplement-8.pdf]

Supplementary Tables

Supplementary Table 1

Tissue	Cases	Cases with CD303$^+$ cells (% total)	pDC Distribution		
			Groups of >20 cells (% total)	Groups of 5-20 cells (% total)	Single cells (% total)
Lung	8	5 (63%)	0	1 (20%)	4 (80%)
Lymph node	8	7 (88%)	3 (43%)	1 (14%)	3 (43%)

Supplementary Table 1. Quantification of the number of human lung and lymph nodes with pDCs in the same field of view as *Mtb* granulomas, as well as enumeration of the pDC distribution. Related to Figure 5.
